# Influence of Defined Hydrophilic Blocks within Oligoaminoamide Copolymers: Compaction versus Shielding of pDNA Nanoparticles

**DOI:** 10.3390/polym9040142

**Published:** 2017-04-19

**Authors:** Stephan Morys, Ana Krhac Levacic, Sarah Urnauer, Susanne Kempter, Sarah Kern, Joachim O. Rädler, Christine Spitzweg, Ulrich Lächelt, Ernst Wagner

**Affiliations:** 1 Department of Pharmacy, Pharmaceutical Biotechnology, Ludwig-Maximilians-Universität München, 81377 München, Germany; stephan.morys@cup.uni-muenchen.de (S.M.); ana.levacic@cup.uni-muenchen.de (A.K.L.); sarah.kern@cup.uni-muenchen.de (S.K.); 2 Department of Internal Medicine IV, University Hospital, Ludwig-Maximilians-Universität München, 81377 München, Germany; sarah.urnauer@med.uni-muenchen.de (S.U.); christine.spitzweg@med.uni-muenchen.de (C.S.); 3 Department of Physics, Ludwig-Maximilians-Universität München, 80799 München, Germany; susanne.kempter@physik.lmu.de; 4 Department of Physics, and Center for NanoScience (CeNS), Ludwig-Maximilians-Universität München, 80799 München, Germany; raedler@lmu.de; 5 Department of Pharmacy, Pharmaceutical Biotechnology, and Center for NanoScience (CeNS), Ludwig-Maximilians-Universität München, 81377 München, Germany; ulrich.laechelt@cup.uni-muenchen.de

**Keywords:** complex stability, nanoparticle, oligoamine, nucleic acid delivery, plasmid DNA (pDNA), polyethylene glycol (PEG), polyplex, shielding

## Abstract

Cationic polymers are promising components of the versatile platform of non-viral nucleic acid (NA) delivery agents. For a successful gene delivery system, these NA vehicles need to comprise several functionalities. This work focuses on the modification of oligoaminoamide carriers with hydrophilic oligomer blocks mediating nanoparticle shielding potential, which is necessary to prevent aggregation or dissociation of NA polyplexes in vitro, and hinder opsonization with blood components in vivo. Herein, the shielding agent polyethylene glycol (PEG) in three defined lengths (12, 24, or 48 oxyethylene repeats) is compared with two peptidic shielding blocks composed of four or eight repeats of sequential proline-alanine-serine (PAS). With both types of shielding agents, we found opposing effects of the length of hydrophilic segments on shielding and compaction of formed plasmid DNA (pDNA) nanoparticles. Two-arm oligoaminoamides with 37 cationizable nitrogens linked to 12 oxyethylene units or four PAS repeats resulted in very compact 40–50 nm pDNA nanoparticles, whereas longer shielding molecules destabilize the investigated polyplexes. Thus, the balance between sufficiently shielded but still compact and stable particles can be considered a critical optimization parameter for non-viral nucleic acid vehicles based on hydrophilic-cationic block oligomers.

## 1. Introduction

In modern cancer therapy, conventional chemotherapy and surgery often fail [1]. Nevertheless, with the field of gene therapy, a novel opportunity has reached clinical application [2,3,4]. Despite all the benefits of viral gene therapy, it also comes with certain disadvantages, such as limited re-administration due to immediate inflammatory or delayed humoral or cellular immune response. In the last decades, much focus has also been put on non-viral gene therapy [5,6]. Polymeric vehicles such as linear polyethylenimine (lPEI) with its repetitive diaminoethane motif can address different stages of the delivery pathway in a way similar to viruses [6,7,8]. Polycations can stably bind and compact pDNA into “polyplexes” [9] with virus-like nanodimensions [10]. Size, stability, and shape of nanoparticles [11,12] play a crucial role for the in vivo biodistribution and survival of nanoparticles, as well as cellular uptake processes [13]. Following cellular uptake by endocytosis, endolysosomal escape of nanoparticles presents a significant bottleneck in the transfection process. lPEI polyplexes are quite effective at this stage due to the endosomal buffering (“proton sponge”) diaminoethane motif [14,15,16,17,18,19]. Nevertheless, the toxicity [20,21] and polydispersity [22] of lPEI led us to the development of diaminoethane motif-containing building blocks suitable for solid phase synthesis (SPS) [23,24]. With the help of SPS, it is possible to synthesize multifunctionalized defined oligomers for gene delivery [25,26,27,28,29,30].

The introduction of a shielding domain into artificial gene carriers is crucial in the first phase of the delivery process to reduce unwanted interaction between blood and pDNA polyplexes. Polyethylene glycol (PEG) represents the most prominent and well-established shielding agent [31,32], and has been used for shielding cationic polyplexes in numerous instances [33,34,35,36,37,38]. It was recently found that PEGylation may cause an immunogenic response [39,40,41,42,43,44]; thus, several alternatives have also been evaluated for this polyplex shielding, such as poly(*N*-(2-hydroxypropyl)methacrylamide) (pHPMA) [45,46,47], hydroxyethyl starch (HES) [48], polysarcosine (pSar) [49,50], oligosaccharides [46,51], or proteins [52]. Recently, recombinant PAS composed of repeats of the natural amino acid sequence proline-alanine-serine was reported as an alternative to PEG [53,54].

Aside from having clearly positive effects such as improved blood circulation times and increased tumor accumulation, polyplex shielding may also provide negative drawbacks, such as a reduced intracellular performance and/or reduced plasmid DNA (pDNA) compaction; this dilemma can be at least partly overcome by programmed deshielding [38,55,56]. The current paper focuses on the relation of polyplex shielding with pDNA compaction, size, shape, and stability. For this purpose, we compare oligoaminoamide co-oligomers with different PEG lengths or defined numbers of PAS repeats which were generated by SPS. We focused on the effect of the shielding block attached in T-shape topology onto a “two-arm” structure of oligoaminoamide oligomers tailored for pDNA delivery. The oligomer library for this study consisted of oligomers decorated with four or eight repetitions of PAS as well as PEG of 12, 24, or 48 ethylene oxide (EO) units in length, or a three-arm oligomer without shielding, respectively. Oligomers were compared and evaluated in different physicochemical and biological assays to elucidate the impact of shielding agent on critical polyplex characteristics.

## 2. Materials and Methods

### 2.1. Materials

2-Chlorotrityl chloride resin was purchased from Iris Biotech (Marktredwitz, Germany), 2-(1*H*-benzotriazol-1-yl)-1,1,3,3-tetramethyluronium-hexafluorophosphate (HBTU), syringe reactors (PP reactor with PE frit) from Multisyntech GmbH (Witten, Germany). All amino acids, peptide grade dimethylformamide (DMF), *N*,*N*-diisopropylethylamine (DIPEA), and trifluoroacetic acid (TFA) were purchased from Iris Biotech (Marktredwitz, Germany). The cationic building block Fmoc-Stp(Boc)_3_-OH (Stp: succinyl-tetraethylene pentamine; Boc: *tert*-butyloxycarbonyl) was prepared as described in [57]. 1-Hydroxy-benzotriazole (HOBt), triisopropylsilane (TIS), dimethylsulfoxide (DMSO), 3-(4,5-dimethylthiazol-2-yl)-2,5-diphenyltetrazolium bromide (MTT), and Triton X-100 were obtained from Sigma-Aldrich (Munich, Germany). *N*-hexane and *tert*-butyl methyl ether (MTBE) were obtained from Brenntag (Mühlheim an der Ruhr, Germany). Dichloromethane was obtained from Bernd Kraft (Duisburg, Germany). Fmoc-*N*-amido-dPEG_12/24_-acid^®^ was purchased from Quanta Biodesign (Powell, OH, USA). 1,2-Ethanedithiol (EDT) was purchased from Sigma-Aldrich (Munich, Germany). All solvents and other reagents were acquired from Sigma-Aldrich (Munich, Germany), Iris Biotech (Marktredwitz, Germany), Merck (Darmstadt, Germany), or AppliChem (Darmstadt, Germany).

All cell culture consumables were obtained from NUNC (Langenselbold, Germany), TPP (Trasadingen, Switzerland), or Sarstedt (Nümbrecht, Germany). DMEM medium was purchased from Sigma Aldrich (Munich, Germany). RPMI medium, fetal bovine serum (FBS), cell culture media, and antibiotics were purchased from Invitrogen (Karlsruhe, Germany), glucose from Merck (Darmstadt, Germany), HEPES from Biomol GmbH (Hamburg, Germany), and sodium chloride from Prolabo (Haasrode, Belgium). Agarose NEEO Ultra-Qualität was obtained from Carl Roth GmbH (Karlsruhe, Germany) and GelRed™ from VWR (Darmstadt, Germany). Heparin sulfate was purchased from Ratiopharm (Ulm, Germany) with 5000 IU/mL. Luciferase cell culture 5× lysis buffer and d-luciferin sodium were obtained from Promega (Mannheim, Germany).

### 2.2. Oligomer Synthesis

Peptide synthesis was carried out using Fmoc-chemistry and l-amino acids. Prior to synthesis, 2-chlorotrityl chloride resins were loaded with the appropriate C-terminal amino acids. Coupling was carried out with 4 eq. of HBTU, 4 eq. of DIPEA, 4 eq. of HOBt, and 4 eq. of Fmoc-protected amino acid per free amine on the solid support; the solution was supplemented with 1% Triton X-100 (*v*/*v*). Amino acids and HOBt were dissolved in NMP (*N*-methylpyrrolidone), supplemented with Triton-X. HBTU was dissolved in DMF, and DIPEA diluted with NMP. Peptide synthesis was carried out using an automated Syro Wave (Biotage, Uppsala, Sweden) with a microwave cavity. Double couplings were performed at 50 °C for 12 min prior to five repetitive washing steps. Fmoc removal was conducted by standard deprotection methods with 40% piperidine in DMF (*v*/*v*) supplemented with 1% Triton-X for 5 × 10 min.

Resins for two-armed oligomers were loaded with Dde-l-Lys(Fmoc)-OH, and for three-arm oligomers with Fmoc-l-Cys(Trt)-OH, as described in the Supplementary Materials. After loading determination and Fmoc removal, Fmoc-Ser(tBu)-OH, Fmoc-Ala-OH, and Fmoc-Pro-OH (from now on called PAS) were attached sequentially, with four or eight triple sequence repeats (PAS_4_, PAS_8_) in the case of PAS-shielded oligomers, while PEG controls were synthesized by attachment of precise bifunctional Fmoc-*N*-amido-dPEG_12_-OH or Fmoc-*N*-amido-dPEG_24_-OH. At this step, an analytical cleavage of a small fraction was done and MALDI-TOF mass spectrometry of the Dde-K-(PAS)_8_ peptide was carried out (see Figure S6).

Next, the cationic backbone of the oligomers was built, based on alternating Stp and histidine units, a lysine branching point, and terminal cysteines for cross-linkage. Therefore, Fmoc-l-His(Trt)-OH, Fmoc-l-Lys(Fmoc)-OH, our cationic building block Fmoc-Stp(Boc)_3_-OH [23], and Boc-l-Cys(Trt)-OH were coupled following the sequence in Table 1. A detailed step-by-step description of the synthesis of Fmoc-Stp(Boc)_3_-OH, of the cationic backbone, and of the three-arm oligomer can be found in [57]. In the case of shielded oligomers, the resins loaded with Dde-l-Lys-PAS_4_/PAS_8_/dPEG_12_/dPEG_24_/dPEG_48_ were placed separately into the microwave cavity of the Syro Wave (Biotage, Uppsala, Sweden) peptide synthesizer, and synthesis was conducted under the conditions described above until the last coupling of *N*-terminal cysteine.

In the case of non-shielded three-arm oligomer, a Cys(Trt)-OH preloaded 2-chlorotrityl resin was used and automated microwave-assisted synthesis was carried out to obtain the sequence mentioned in Table 1. All oligomers were ended by a double coupling of Boc-Cys(Trt)-OH for 2 × 1 h at room temperature to avoid racemization.

Synthesis of cMet-targeted oligomers (containing a cMet-binding peptide, cmb) can be found in the experimental section of Supplementary Materials.

After the final coupling step, the resins were dried and the products cleaved off the resin for 90 min using a mixture of TFA/EDT/H_2_O/TIS 94:2.5:2.5:1 (*v*/*v*) at a ratio of 10 mL per gram resin.

The cleaved oligomers were purified by size exclusion chromatography (SEC) performed with 10 mM hydrochloric acid/acetonitrile 7:3 as solvent. An ÄKTA purifier system (GE Healthcare Biosciences, Uppsala, Sweden) equipped with a Sephadex G-10 column and a P-900 solvent pump module, a UV-900 spectrophotometrical detector, a pH/C-900 conductivity module, and a Frac-950 automated fractionator was used. The product fractions were collected and combined prior to lyophilisation.

Analytical data of ^1^H-NMR and reversed phase high-performance liquid chromatography (RP-HPLC) can be found in Figures S7–S30.

### 2.3. Polyplex Formation

Indicated amounts of pCMVLuc (CMV: cytomegalovirus promotor; Luc: firefly luciferase gene) and oligomer at indicated nitrogen/phosphate (N/P) ratios were diluted in separate tubes. If not otherwise mentioned, suspension media was 20 mM HEPES buffered 5% glucose at pH 7.4 (HBG 7.4). Diluted oligomer and diluted pDNA were always mixed at similar volume.

For N/P ratio calculation, only the protonatable amino groups of the Stp units and *N*-terminal amines of cysteine residues were considered (N number) and related to the anionic phosphate groups (P number) in pDNA. Note that protonatable amines outside of the cationic backbone and protonatable imidazole nitrogens of histidines were not taken into account for calculation of N (for reasons of consistency with our previous work). The nucleic acid was added to the oligomer, mixed vigorously, and incubated for 30 min at room temperature under air exposure to enhance disulfide formation.

### 2.4. Polyplex Stability in the Presence of Salt

Polyplexes were prepared with oligomers at N/P 12 and 2 µg pCMVLuc in a total volume of 60 µL deionized water. After 30 min, 500 µL of phosphate-buffered saline (PBS) was added and a dynamic laser-light scattering (DLS) measurement with three runs (including six sub-runs each) was performed immediately. Samples were incubated at room temperature, and further measurements after 5, 30, 60, and 180 min were conducted.

### 2.5. Erythrocyte Adhesion in the Presence or Absence of Serum

Polyplexes were prepared at N/P 12 and 2 µg pDNA (20% Cy5 labeled) in a total volume of 60 µL HBG. After 30 min, three groups were treated differently. Either HBG, 3 × 10^6^ erythrocytes in HBG, or serum (to a final concentration of 90%) were added. After further 30 min of incubation at 37 °C, erythrocytes containing polyplexes were sedimented by centrifugation (1500 rpm for 10 min at room temperature) and the supernatant was taken. Then, 3500 IU of heparin sulfate was added to dissociate the polyplex and determine the remaining amount of pDNA via Cy5 excitation/emission (λ_ex_ = 649 nm and emission wavelength λ_em_ = 670 nm). Data were calculated in comparison to equally treated free pDNA. Self-quenching and efficacy of release with 3500 IU heparin can be found in Figure S1.

### 2.6. Particle Size and Zeta Potential

Particle size and zeta potential of polyplexes were measured by dynamic laser-light scattering using a Zetasizer Nano ZS (Malvern Instruments, Worcestershire, UK) and transmission electron microscopy (TEM) with a JEM 1011 (Jeol, Freising, Germany).

For DLS measurements, 2 µg pDNA was dissolved in 30 µL HBG and was added to an amount of oligomers corresponding to N/P 12 in 30 µL HBG. After 30 min of incubation at room temperature, 740 µL of 10 mM sodium chloride solution (pH 7.4) was added and the samples were measured. Results were plotted as Z-Average and SD out of three runs with 12 sub-runs each. Zeta potential (ZP) is displayed as average (mV) of three runs with up to 15 sub-runs each.

For TEM, samples were prepared as follows. The formvar/carbon-coated 300 mesh copper grids (Ted Pella Inc., Redding, CA, USA) were activated by mild plasma cleaning. Afterwards, the grids were incubated with 20 µL of the polyplex solution at N/P 12 for 2.5 min previously prepared in water. Excess liquid was blotted off using filter paper until the grid was almost dry. Prior to staining, the grids were washed with 5 µL of staining solution for 5 s. Then, the copper grids were incubated with 5 μL of a 2% aqueous uranylformate solution for 20 s, excess liquid was blotted off using filter paper, followed by air-drying for 30 min. Samples were then analyzed using a JEM 1011 (Jeol, Freising, Germany) operated at 80 kV.

### 2.7. Ethidium Bromide Compaction Assay and Heparin Stress

Polyplexes containing 2 μg pDNA were formed at N/P ratios of 6 and 12 in a total volume of 200 μL HBG. lPEI polyplexes formed at N/Ps 6 and 12 served as a positive control. HBG buffer (200 μL) served as blank, and 2 μg pDNA in 200 μL HBG buffer was considered as maximum ethidium bromide (EtBr) fluorescence intensity (100% value). These samples were prepared in parallel to the polyplexes. After 30 min of incubation at room temperature, 700 μL of EtBr solution (*c* = 0.5 μg/mL) were added. The fluorescence intensity of EtBr was measured after an additional 3 min incubation using a Cary Eclipse spectrophotometer (Varian, Germany) at the excitation wavelength λ_ex_ = 510 nm and emission wavelength λ_em_ = 590 nm. The fluorescence intensity of EtBr was determined in relation to the 100% value. As a further experiment, 250 IU of heparin (Ratiopharm, Ulm, Germany) was added to the samples after EtBr addition to investigate polyplex stability against polyanionic stress.

### 2.8. Serum Stability of Polyplexes

Determination of polyplex stability against 90% FBS was determined by DLS. Polyplexes were prepared as described previously with 8 µg pDNA and oligomers at N/P 12 in a total volume of 50 µL HBG. After incubation for 30 min, 30 µL of HBG and 720 µL FBS were added to reach a final concentration of 90% FBS. Sixty microliters were placed in a DTS1070 cuvette, and *t* = 0 min was determined. Then, polyplexes in serum were incubated under steady shaking at 37 °C and aliquots were taken for further measurements after 30, 120, 240 min, and 24 h. Each time point represents one measurement averaged from 15 sub runs.

### 2.9. Cell Culture

A mouse neuroblastoma cell line (Neuro-2a) as well as the human hepatocellular carcinoma cell line Huh7 were cultured in Dulbecco’s Modified Eagle’s Medium (DMEM) (1 g/L glucose), while human prostate cancer cell line (DU145) was cultured in RPMI-1640 medium. All media were supplemented with 10% fetal bovine serum, 4 mM stable glutamine, 100 U/mL penicillin, and 100 μg/mL streptomycin. Cell lines were cultured at 37 °C and 5% CO_2_ in an incubator with a relative humidity of 95%.

### 2.10. In Vitro Gene Transfer

Cells were seeded 24 h prior to pDNA delivery in 96-well plates. For transfections of Neuro-2a and DU145, 10,000 cells/well were seeded, and 8000 cells/well were seeded in the case of Huh7. Transfection efficiency of oligomers was evaluated using 200 ng pCMVLuc per well. Polyplexes were formed at different N/P ratios in a total volume of 20 μL HBG. Before treatment, the cell culture medium was exchanged with 80 μL fresh medium containing 10% FBS. Polyplex solution was added to each well and incubated on cells at 37 °C for a determined period of time (45 min or 24 h). In the first case, medium was replaced 45 min after transfection by fresh medium. For control experiments, only fresh medium was added. In the second case, cells were incubated with polyplex solution for 24 h after initial transfection. All experiments were performed in quintuplicate. lPEI (N/P 6) was used as a positive control, and HBG buffer was used as a negative control.

For the luciferase assay, 24 h after initial transfection, medium was removed and cells were treated with 100 μL luciferase cell culture 5× lysis buffer. Luciferase activity in the cell lysate was measured by using a Centro LB 960 plate reader luminometer (Berthold Technologies, Bad Wildbad, Germany) and LAR buffer supplemented with 1 mM luciferin. Transfection efficiency was evaluated as relative light units (RLU) per well.

For the metabolic activity of transfected cells, 24 h after initial transfection, 10 µL of MTT was added to each well, reaching a final concentration of 0.5 mg/mL. Medium with unreacted dye was removed after an incubation time of 2 h at 37 °C. The 96-well plates were stored at −80 °C for at least one hour, and afterwards the purple formazan product was dissolved in 100 µL DMSO per well. The absorbance was determined by using a microplate reader (TecanSpectrafluor Plus, Tecan, Switzerland) at 530 nm with background correction at 630 nm. The relative cell viability (%) related to the buffer-treated control cells was calculated as ([A] test/[A] control) × 100%.

### 2.11. Cellular Association

Neuro-2a cells were seeded 24 h prior to transfection into 24-well plates at a density of 50,000 cells/well. Culture medium was replaced with 400 μL fresh growth medium 24 h after seeding the cells. pDNA polyplexes formed at N/P ratio 12 in 100 μL HBG and containing 1 μg pCMVLuc (20% of Cy5-labeled pCMVLuc) were added to each well and incubated on ice for 30 min. Subsequently, cells were washed twice with 500 µL PBS. Cells were detached with trypsin/EDTA and resuspended in PBS with 10% FBS. All experiments were performed in triplicate. Cellular association of the polyplexes was measured by excitation of Cy5 at 635 nm and detection of emission at 665 nm. DAPI (4′,6-diamidino-2-phenylindole) staining was used to discriminate between viable and dead cells. Cells were properly gated by forward/sideward scatter and pulse width for the exclusion of doublets. Data were recorded by Cyan ADP flow Cytometer (Dako, Hamburg, Germany) using Summit acquisition software (Summit, Jamesville, NY, USA) and analyzed by FlowJo 7.6.5 flow cytometric analysis software.

### 2.12. Cellular Internalization

Neuro-2a cells were seeded 24 h prior to transfection into 24-well plates at a density of 50,000 cells/well. Culture medium was replaced with 400 μL fresh growth medium 24 h after seeding the cells. pDNA polyplexes formed at N/P ratio 12 in 100 μL HBG and containing 1 μg pCMVLuc (20% of Cy5-labeled pCMVLuc) were added to each well and incubated at 37 °C for 45 min. Subsequently, cells were washed once with 500 µL PBS containing 1000 IU heparin for 15 min on ice to remove any polyplexes sticking to the cell surface and again washed once with 500 μL PBS only. Cells were detached with trypsin/EDTA and resuspended in PBS with 10% FBS. All experiments were performed in triplicate. Cellular internalization of the polyplexes was measured by the excitation of Cy5 at 635 nm and detection of emission at 665 nm. DAPI staining was used to discriminate between viable and dead cells. Cells were properly gated by forward/sideward scatter and pulse width for exclusion of doublets. Data were recorded by Cyan ADP flow Cytometer (Dako, Hamburg, Germany) using Summit acquisition software (Summit, Jamesville, NY, USA) and analyzed by FlowJo 7.6.5 flow cytometric analysis software.

### 2.13. In Vivo Gene Transfer

Animal experiments were performed in female 6-week-old nude mice, Rj: NMRI-nu (nu/nu) (Janvier, Le-Genest-St-Isle, France) which were housed in isolated ventilated cages with a 12 h day/night interval and food and water ad libitum. Huh7 (5 × 10^6^ cells) suspended in 150 μL PBS were injected subcutaneously into the left flank. After injection, tumor size was monitored with a caliper and determined by formula a × b^2^/2 (a = longest side of the tumor; b = widest side vertical to a). When tumors reached a size of approximately 1200 mm^3^, the experiments started by intratumoral injection of 60 μL polyplex solution containing 50 μg pCMVLuc at N/P 12 in HBG. For each polymer, a group of five mice (*n* = 5) was treated. Mice were euthanized 48 h later, and tumors were collected to assess luciferase activity via ex vivo luciferase assay. Tumors were homogenized in 500 μL cell lysis buffer using a tissue and cell homogenizer (FastPrep^®^-24). To separate insoluble cell components, the samples were centrifuged at 3000× *g* at 4 °C for 10 min. Luciferase activity was measured in the supernatant using a Centro LB 960 luminometer. All animal experiments were performed according to the guidelines of the German law for the protection of animal life, and were approved by the local animal ethics committee.

### 2.14. Statistical Analysis

Statistical significance was analyzed by Student’s one-tailed *t*-test. Significance levels are indicated with symbols: ns *p* > 0.05; * *p* ≤ 0.05; ** *p* ≤ 0.01; *** *p* ≤ 0.001; **** *p* ≤ 0.0001.

## 3. Results and Discussion

### 3.1. Peptide and Oligomer Synthesis

**Scheme 1:**
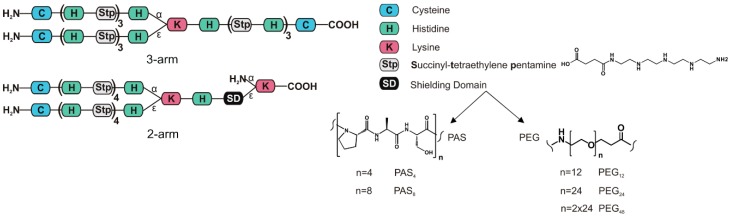
Schematic structures of the oligomer topologies evaluated in this paper. PAS: proline-alanine-serine; PEG: polyethylene glycol.

The objective of this study was to examine different hydrophilic blocks—PAS and PEG—with varying length in combination with a cationic oligoaminoamide backbone for their “shielding abilities” in pDNA polyplexes. The cationic oligomers can mainly be divided into two subgroups: a three-arm cationic topology without shielding domain [58] for control carriers, or shielded two-arm structures comprising certain PEG or PAS repetitions—topologies are described in Scheme 1. PAS represents the three neutral amino acids proline, alanine, and serine, ordered from N to C terminus. Solid phase assisted synthesis (SPS) was used for the assembly of the oligomers listed in Table 1. This method allowed oligomers to be easily tailored to our needs. The non-shielded positive control with three-arm topology had a similar number of charge-bearing units (9 Stp, 12 histidines) as the shielded two-arm oligomers (8 Stp, 11 histidines). A two-arm structure without shielding agent might have been considered as a suitable control, but previous studies [24] revealed a lower efficacy of short cationic two-arm oligomers. Moreover, a shielded two-arm in fact presents a three-arm topology (with the shielding polymer block arm representing the third arm).

The length of shielding blocks used in this study were chosen along the commercially available, precise PEG derivates of 12 or 24 ethylenoxide (EO) units and analogous PAS peptides. So PAS_4_ consisting of four PAS repeats (4 × 3 = 12 amino acids) was considered as backbone analog to 12 EO units (PEG_24_), and PAS_8_ (8 × 3 = 24 amino acids) as analog to 24 EO units (PEG_24_). Due to the amino acid side groups, the molecular weight of a PAS tri-amino acid block is substantially higher (273 g/mol) than the analogous EO trimer block (3 × 44 g/mol = 132 g/mol).

In case of shielded two-arm structures, first a Dde-l-Lysine-(Fmoc) was loaded on a 2-chlorotrityl resin. Consequently, the shielding domain was attached to the ε-amine after successful Fmoc deprotection. It consisted of either monodisperse polyethylene glycol (PEG) of 12, 24, or 2 × 24 units, or of four or eight repetitive Ser–Ala–Pro units. The cationic backbone consists of histidines, the artificial amino acid Stp (succinyl-tetraethylene pentamine) [23], lysine, and cysteine. Histidines were introduced for enhanced endosomal buffering [25,29,59]. Stp was used for nucleic acid packaging, endosomal buffering, and endosomal escape [58,60]. The diamino acid lysine served as a branching point, and *N*-terminal cysteines were introduced for polyplex stabilization via disulfide-crosslinking [24,58,61]. Each oligomer was characterized by ^1^H-NMR and RP-HPLC. Analytical data can be found in Figures S7–S30.

### 3.2. Physicochemical Polyplex Characterization

Oligomer/pDNA interaction was examined in different assays, focusing on pDNA binding abilities, stability, and compaction. First, pDNA binding potency of the oligomers was investigated by agarose gel electrophoresis shift assays. Here polyplexes were formed with different ratios of oligomer to pDNA. This ratio is displayed as N/P, with varying amount of oligomer but constant amount of 200 ng pDNA (see Figures S2 and S3). This assay revealed that all tested oligomers efficiently complex pDNA at an N/P ratio between 2 and 2.5. We already reported similar findings with oligomers composed of the same cationic backbone [25,26]. Importantly, the presence and nature of shielding elements did not influence the pDNA binding of the cationic backbone in this assay for shielding agents with the length up to PEG_24_. Only the oligomer with longest PEG_48_ shielding domain exhibited slightly decreased binding potency, since certain fractions of free pDNA could still be observed at N/P 3 and 6. Notably, pDNA binding does not necessarily correlate with its degree of compaction or polyplex shape. Polyplex properties regarding DNA compaction were therefore investigated with alternative techniques (see Section 3.4).

After pDNA binding was confirmed, particle sizes and zeta potential were determined by dynamic light scattering at N/P 12. Results revealed very homogenous particles with a polydispersity index (PDI) between 0.04 and 0.33, where 1.0 represents the highest polydispersity (see Table 2). All polyplexes show D_H_ values (displayed as Z-Average in nm) in the range between 98 nm and 147 nm. It has been reported that particles up to 200 nm can be taken up via a clathrin-dependent pathway [62], indicating that subsequent uptake into cells should be possible via endocytosis. DLS data provide a hint that particles with shorter shielding agents (PEG_12_ or PAS_4_) exhibit smaller particle sizes than longer shielding agents. This could be explained by more compact and condensed particles.

### 3.3. Steric Shielding

**Figure 1:**
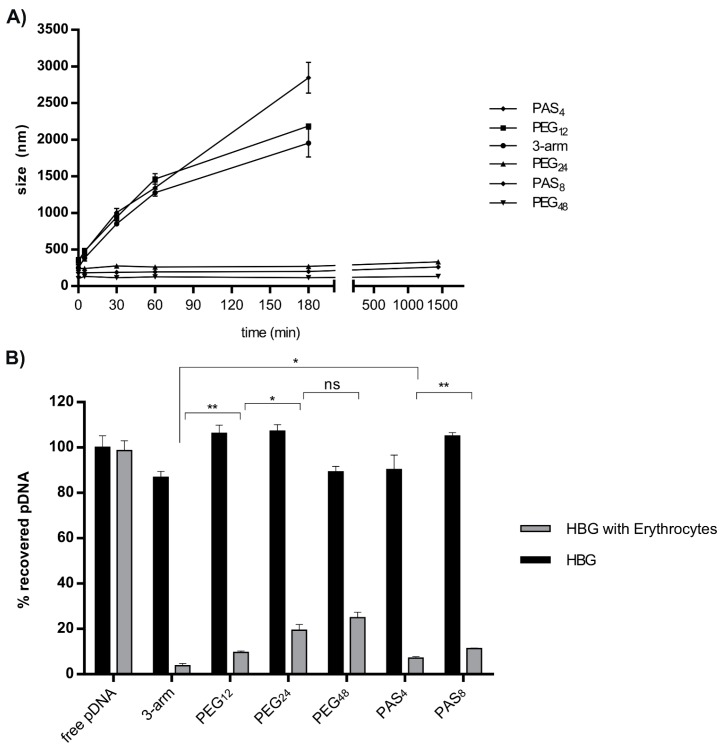
(**A**) Stability testing of untargeted polyplexes against phosphate-buffered saline (PBS). After 30 min of polyplex incubation (2 µg pDNA; N/P 12) in deionized water and addition of 500 µL PBS, DLS measurement was performed. Time points 0, 5, 30, 60, and 180 min were displayed. In case of colloidally-stable polyplexes of PEG_24_, PEG_48_, and PAS_8_, a measurement after 24 h is also displayed. Data are presented as mean value (±SD) out of triplets; (**B**) Polyplexes (2 µg pDNA; N/P 12) were incubated in HBG with or without 3 × 10^6^ erythrocytes. After centrifugation, the supernatant was taken, and 3500 IU of heparin were added to release pDNA of the polyplexes. Cy5 fluorescence was compared to uncomplexed (free) pDNA. Statistical analysis (Student’s *t*-test): ns *p* > 0.05, * *p* ≤ 0.05; ** *p* ≤ 0.01.

As a next step to characterize polyplexes, a stress assay was performed with salt to evaluate the behavior in isotonic salt concentration. Therefore, PBS buffered at pH 7.4 was added to the polyplexes after 30 min of incubation in deionized water. DLS measurements were performed immediately after addition, and at 5, 30, 60, and 180 min. As displayed in Figure 1A, the unshielded three-arm started to aggregate within 5 min after addition of PBS. Along with the three-arm, PAS_4_ and PEG_12_ also underwent colloidal aggregation. Meanwhile PEG_24_-, PEG_48_-, and PAS_8_-decorated polyplexes showed colloidal stability over 24 h without any significant aggregation. This indicates that longer PEG or PAS chains provide improved colloidal stability with lower risk for aggregation, while polyplexes with shorter PEG length or lack of shielding agent show immediate aggregation after salt addition. These findings (together with the DNA binding studies) are in accordance with previously published work where PEG shielding of PEI polyplexes increased the colloidal stability of polyplexes against salt-induced stress, however at the expense of reduced pDNA binding [63,64].

The next experiment was designed to investigate the interaction of polyplexes with erythrocytes. Results are displayed in Figure 1B. Polyplexes in HBG and polyplexes after incubation with erythrocytes were dissociated by heparin addition, and Cy5 emission of released labeled pDNA was detected. pDNA can be fully released by heparin (Figure 1B, dark bars) within the range of experimental accuracy. The data reveal a reduced pDNA recovery due to erythrocyte binding (grey bars); they also reveal that interaction with erythrocytes decreases with ascending length of the shielding agents. PEGylation is known to reduce the interaction of polyplexes with erythrocytes [63], which is in accordance with our findings.

### 3.4. DNA Compaction

**Figure 2:**
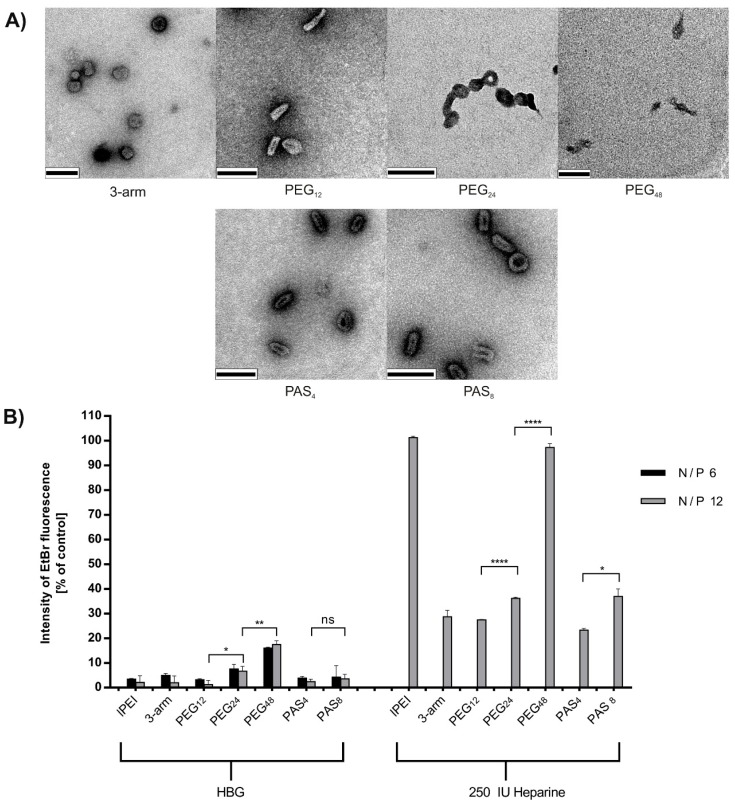
(**A**) Transmission electron microscopy images of polyplexes formed at N/P 12 in deionized water and stained with uranylformate. Scale bar represents 100 nm; (**B**) **Left**: pDNA compaction, correlating with the remaining fluorescence of ethidium bromide (EtBr). Results are calculated against free pDNA. **Right**: Polyplexes at N/P 12 after addition of 250 IU of heparin. lPEI: linear polyethylenimine. Statistical analysis (Student’s *t*-test): ns *p* > 0.05, * *p* ≤ 0.05; ** *p* ≤ 0.01; **** *p* ≤ 0.0001.

Transmission electron microscopy (TEM) was performed to investigate polyplex size and shape. While differences between shielding agents could not be observed by TEM, we observed a clear difference compared to D_H_ in general, but more unexpected between polyplexes decorated with shorter or longer shielding agents (Figure 2A). Particles formed out of oligomers with shorter shielding agents tend to be not only smaller, but also more condensed regarding their structure compared to polyplexes shielded with longer shielding agents. Polyplexes with PEG_12_ or PAS_4_ exhibited sizes of around 50 nm and a more condensed “rod-like” structure, while PEG_24_- or PAS_8_- decorated polyplexes revealed sizes of approximately 80–100 nm in a “doughnut-like” state. PEG_48_-decorated polyplexes revealed the lowest level of compaction and a very erratic shape of around 100 nm in size. Polyplexes including no shielding agent displayed the most condensed population with sizes around 40–50 nm in a very compact globular shape. However, polyplexes tend to aggregate due to their charged surface; this effect is not visible in case of polyplexes decorated with short shielding agents. These findings are in accordance with recent observations [38], where particle morphology changed from long rods to globular condensed polyplexes with decreasing amounts of conjugated PEG. To further investigate the ability to compact pDNA depending on the shielding agent used, pDNA compaction was determined with an ethidium bromide (EtBr) exclusion assay (Figure 2B). Therefore, 2 µg of pDNA was mixed with oligomers at either N/P 6 or 12, incubated for 30 min, and then measured after the addition of EtBr. As reference, linear PEI was included in this study. The intensity of EtBr fluorescence normalized to uncomplexed pDNA is displayed in Figure 2B (left). These data support the findings from previous agarose gel shifts and confirm the findings from TEM images. All oligomers complex pDNA well, but with increased length of shielding agent, pDNA compaction decreased, which can be seen most pronounced in the case of the longest shielding element PEG_48_. In the right part of Figure 2B, fluorescence of the released pDNA of polyplexes is displayed after the addition of 250 IU of heparin sulfate to cause anionic dissociative stress. Here it can be seen that polyplexes formed with lPEI and polyplexes decorated with PEG_48_ completely release pDNA and cause full EtBr fluorescence, while all other polyplexes are at least partly resistant to the applied heparin stress. Most interestingly, the shorter the PEG respectively PAS shield, the more resistant the polyplexes were towards heparin stress. In other words, long PEG (24 or 48 units) or eight PAS repeats tend to destabilize the polyplex. These results suggest that the cationic charge density of the backbone is just one critical point for nucleic acid interaction, but the length of the shielding agent also plays a certain role. These findings from EtBr exclusion assay confirm our results from TEM. They are also in accordance with a most recent report on lPEI polyplexes [65], where PEGylation caused more sensitivity towards heparin stress as well as lower compaction in general.

### 3.5. Serum Stability

**Figure 3:**
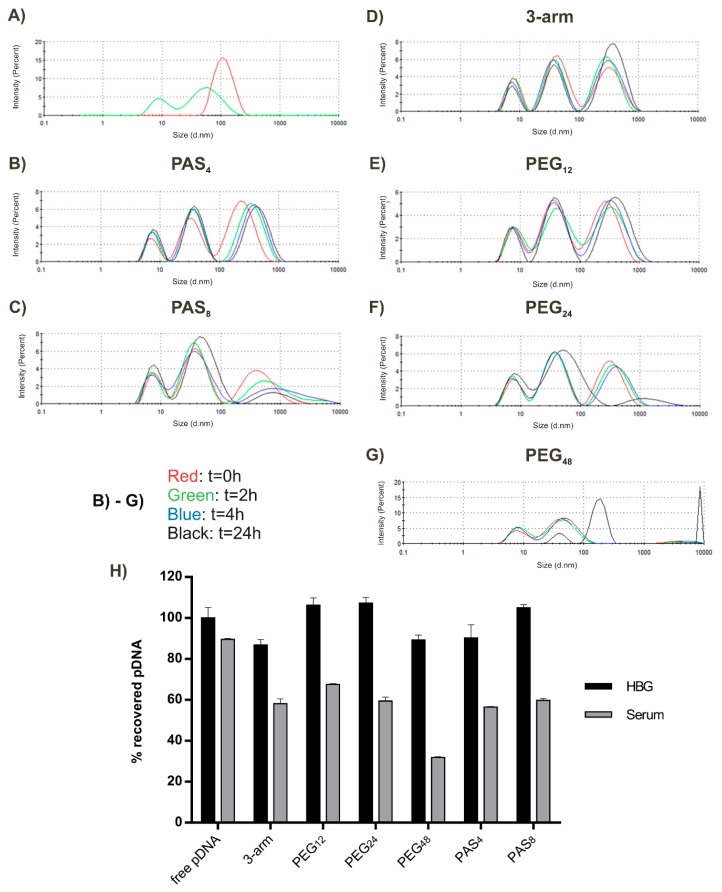
(**A**) DLS measurements of 90% fetal bovine serum (FBS, green) and pDNA polyplexes of the three-arm oligomer in HBG (red) as references to discriminate polyplex and serum peaks; (**B**–**G**) Display behavior of polyplexes with indicated oligomers in 90% FBS over time. In (**H**), the amount of recovered pDNA of polyplexes containing Cy5-labeled pDNA after treatment with 3500 IU heparin is displayed after incubation with FBS for 30 min; HBG-treated polyplexes served as control.

Stability of polyplexes in the blood protein environment presents a most relevant and critical issue, as interaction with proteins can cause aggregation or partial to complete dissociation of the polyplex. Thus, further experiments were designed to investigate polyplex stability in serum. We applied an assay to measure polyplex size in 90% FBS with DLS. This method offered the possibility to monitor polyplex behavior over a long time period from the point of serum addition up to 24 h. Time points *t* = 0, 2, 4, 24 h are displayed in Figure 3B–G. Firstly, the size of all polyplexes increased in serum, from 100–150 nm in 10 mM NaCl to 250–300 nm immediately after addition of FBS. From here on, a decay (loss of intensity and increase in size) of the polyplexes was observed over time in the case of the longer shielding agents PEG_24_ and PAS_8_, with a tendency towards a higher colloidal stability of PAS_8_. PEG_12_ and PAS_4_ were the most promising candidates, with almost no decay over time. While polyplexes shielded with PEG_48_ underwent immediate decay and aggregation, unshielded three-arm underwent no additional changes after the initial size increase within the following 24 h. An explanation for the elevated stability of three-arm, PAS_4_, and PEG_12_ might be the formation of a serum corona around the polyplexes (which also explains the increased size), and due to the higher compaction, the ionic complexes remain stable despite serum interaction. Serum corona—especially when attached to PEGylated nanostructures—has been demonstrated to mediate favorable shielding properties [66].

In Figure 3H, the behavior of polyplexes after 30 min of incubation with 90% FBS is compared to polyplexes maintained for the same time in HBG. The amount of released Cy5-labeled pDNA after addition of heparin was determined. In all cases, pDNA release was lower after polyplex incubation with FBS. In the case of PEGylated polyplexes, a trend towards less pDNA release could be observed with increasing PEG length. At least at this early time point, the formation of a serum corona seems to mediate increased resistance towards polyanionic stress. However, this protective effect seems to reverse when polyplexes are incubated over a long time in serum, maybe due to the lack of compaction (see Figure 3C,F,G).

### 3.6. Tumor Cell Interactions In Vitro

**Figure 4:**
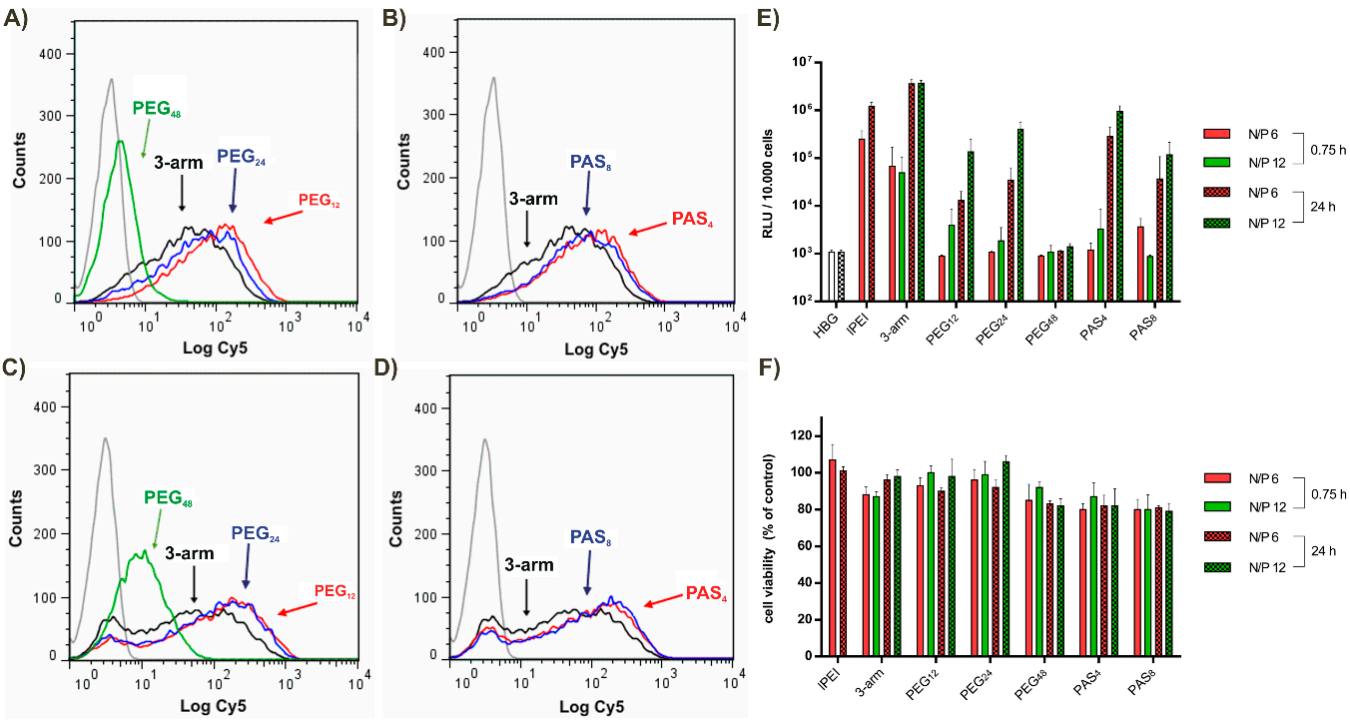
In (**A**,**B**) cellular association of polyplexes with N2a cells after 30 min incubation at 4 °C as determined by flow cytometry is plotted; In (**C**,**D**) cellular internalization of polyplexes after 45 min incubation at 37 °C followed by removal of extracellularly-bound polyplexes is displayed; Logarithmic X-scale in (**A**–**D**) represents Cy5 fluorescence of polyplexes; (**E**) Luciferase reporter gene expression in N2a cells after 0.75 h (without pattern) and after 24 h (with pattern). Transfections were performed at two different ratios: N/P = 6 (red) and N/P = 12 (green). (**F**) Cell viability assay was performed in parallel.

Different interactions of our polyplexes were investigated on N2a (mouse neuroblastoma) cells. In Figure 4A,B cellular binding is shown. Only polyplexes decorated with PEG_48_ showed strong shielding against unspecific binding within 30 min of interaction with cells.

Figure 4C,D displays cellular uptake within 45 min of incubation on cells. Again, only PEG_48_ polyplexes did not interact with cells and did not mediate significant uptake. In Figure 4E, gene transfer activity is shown after 0.75 h and 24 h at N/P ratios 6 and 12. After the early 0.75 h time point, only unshielded three-arm and lPEI polyplexes caused notable gene transfer, while after 24 h of incubation, all polyplexes showed similar transfection efficacy; it is noteworthy that they were as efficient as lPEI. None of the polyplexes mediated cytotoxicity at any time point (see Figure 4F). These results suggest that rapid binding and cellular uptake takes place only in case of unshielded cationic polyplexes (three-arm, lPEI) with sufficient critical endosomal amount of cationic proton sponge carrier required for efficient endosomal escape and transfection [14,15,67]. In case of oligoaminoamide carriers with short shielding elements, the internalized amounts of polyplexes increases over time, reaching sufficient levels for endosomal escape. Only for PEG_48_ polyplexes, notable transfection is not even reached after 24 h. Either the critical amount for endosomal escape is not reached, or the longer PEG domain hampers the endosomal membrane disruption. The widely known effect that introduction of PEG into cationic transfection complexes can reduce transfection efficacy has been referred to as the “PEG dilemma” [56]. For PEI-type polyplexes, it has been assumed that a combined effect of osmotic endosomal burst and direct phospholipid destabilization by the cationized carrier is required for endosomal escape [6,18,68,69]; obviously, PEGylation can hinder the direct cationic membrane destabilization. Consistently, bioreversible PEGyation was demonstrated to resolve this dilemma [55,56,70].

### 3.7. Tumor Cell Interactions In Vitro without and with Targeting

For this study, a phage-derived peptide (cmb) binding specifically to the HGF (hepatocyte growth factor)-receptor cMet [71,72] was introduced into our oligomers via modification at the α-amine of the c-terminal lysine. A complete list of cmb-containing oligomers can be found in Table S1. cMet is widely overexpressed in epithelial-derived tumors as well as stromal and interstitial cell-derived tumors (e.g., sarcomas) [73], and was pointed out previously as a suitable target for tumor targeting [72,74,75]; introduction of cmb into sequence-defined oligomers improved transfection efficacy over untargeted controls in vitro as well as in vivo [25,74].

**Figure 5:**
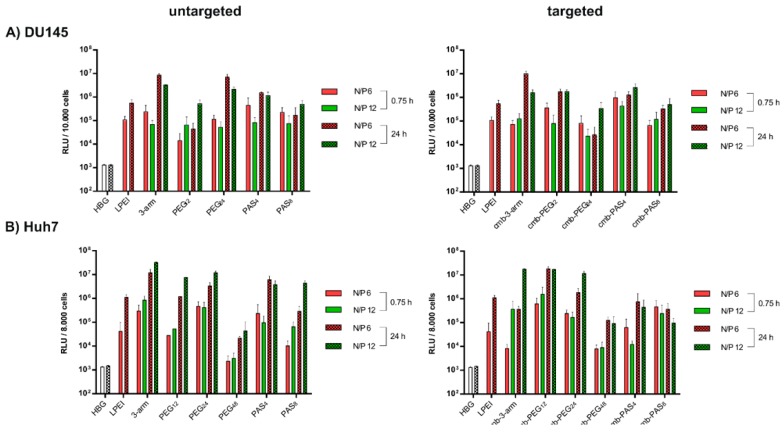

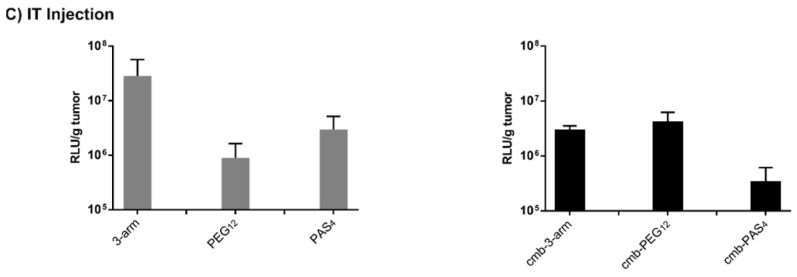
(**A**) Luciferase reporter gene expression in human prostate cancer cell line DU145 as well as in (**B**) human hepatocellular cancer cells Huh7 after 0.75 h (without pattern), and after 24 h (with pattern). Transfections were performed at two different ratios: N/P = 6 (red) and N/P = 12 (green); (**C**) Luciferase gene expression at 48 h after intratumoral administration of pCMVLuc polyplexes at N/P = 12 into Huh7 tumor-bearing mice. Luciferase gene expression is presented as relative light units per gram tumor (RLU/g tumor; *n* = 5, mean ± SEM). Lysis buffer RLU values were subtracted.

In the current work, transfections of two cMet–overexpressing cell lines—the DU145 prostate cancer cell line (Figure 5A) and the Huh7 hepatocellular cancer cell line (Figure 5B)—were carried out with polyplexes without or with cMet targeted ligands. With both cell lines, results were consistent with the previous N2a transfections; short-term incubations with polyplexes for 45 min only mediated moderate gene transfer, while higher gene expression was observed after a longer transfection time of 24 h. Transfection of Huh7 with cmb-PAS_8_ (no increase with time) presents an exemption from this rule. In case of the PEG_12_ containing polymer, we observed an elevating effect of the introduction of the cmb targeting peptide on transfection efficacy. Further on, transfection efficacy of PEG_12_ decorated polyplexes tended to be higher than with PEG_24_-decorated polyplexes. This increased transfection efficacy could be affiliated with the beneficial compact, rod-like structure of PEG_12_-containing polyplexes [76]. MTT assays for both cell lines were also performed in parallel to transfections, and did not show notable cytotoxicity at any time point (see Figure S4). In addition to the transfections of cMet-positive cell lines, transfections of receptor negative N2a cells (for 0.75 h and 24 h) with targeted polyplexes can be found in Figure 5SA. No significant gene transfer was observed after 0.75 h. After 24 h, polyplexes had been taken up unspecifically and caused notable gene transfer in the case of cmb-3-arm, cmb-PEG_12_, and cmb-PEG_24_. cmb-PAS_4_ and cmb-PAS_8_ hardly mediated any gene transfer. Cytotoxicity was not observed (Figure 5SB).

### 3.8. Tumor Cell Interactions In Vivo without and with Targeting

An in vivo experiment was the next logical step to further evaluate the potency of our oligomers. As a first step, intratumoral application into a subcutaneous Huh7 mouse model was chosen. Kos et al. [25] pointed out in the same model that polyplexes formed with cmb-PEG24 were superior to cmb-PEG48 in gene transfer. Therefore, in the current experiments, untargeted as well as targeted polyplexes of 3-arm, PEG12, and PAS4 were intratumorally injected into mice.

Untargeted, unshielded 3-arm mediated the highest gene transfer, as already observed in vitro (see Figure 4E and Figure 5A,B). This might be explained by the local stickiness of unshielded surface and in the tumor. At the same time, polyplexes with PEG12 mediated decreased transgene expression, indicating a successful shielding in vivo. By introduction of targeting peptide cmb into PEG12-shielded polyplexes, transfection efficacy was recovered again. Overall, luciferase gene expression was also increased as compared to cmb-PEG24 [25], which extends the already previously observed trend of increasing gene transfer activity with decreasing PEG length (PEG48 < PEG24 < PEG12). Interestingly, recent work with lPEI polyplexes [65] also reported favorable properties for incorporating targeting peptides via a 500–700 Da PEG molecule, which has similar size as PEG_12_.

## 4. Conclusions

For this study, we synthesized pDNA polyplexes decorated with highly defined PEG as well as PAS repetitions for surface shielding. We compared them side-by-side and with an unshielded control oligomer. Firstly, PAS_4_- and PAS_8_-decorated sequence-defined oligo(ethanamino)amides can be synthesized by SPS and used for the formation of shielded pDNA. Within the performed in vitro structure–activity relationship studies, polyplexes shielded with PEG and PAS of comparable length exhibited similar properties. This finding remains to be extended by in vivo pharmacokinetic experiments investigating systemic circulation and biodistribution. Independent of the hydrophilic block, the length had clear opposite effects on shielding and compaction of formed pDNA nanoparticles. In sum, it is notable that opposing requirements have to be dealt with balanced measures; both extremes (no shielding or very long shielding) have their drawbacks. The oligomer characteristics are summarized in Table 3. In the current study with two-arm oligoaminoamide pDNA polyplexes, the shorter PEG_12_ domain is considered to be the best compromise. Similar conclusions have been recently reported by other researchers both for shielded lPEI polyplexes [65] and liposomes [77]. These findings were also confirmed for polymers containing the targeting peptide cmb. The cmb-PEG_12_ shielded polyplexes showed the most pronounced effect of cMet targeting and mediated the most potent gene transfer in vitro as well as in vivo.

## Figures and Tables

**Table 1:** List of oligomers included in this study. Internal polymer numbers, structures from N to C terminus, as well as abbreviations used within the paper.

Polymer #	Structure	Abbreviation
689	[C-(H-Stp)_3_-H]_α,ε_-K-H-(Stp-H)_3_-C	3-arm
1088	{[C-(H-Stp)_4_-H]_α,ε_-K-H-dPEG_12_}_ε_-K	PEG_12_
1091	{[C-(H-Stp)_4_-H]_α,ε_-K-H-dPEG_24_}_ε_-K	PEG_24_
1120	{[C-(H-Stp)_4_-H]_α,ε_-K-H-dPEG_24_-dPEG_24_}_ε_-K	PEG_48_
1094	{[C-(H-Stp)_4_-H]_α,ε_-K-H-(PAS)_4_}_ε_-K	PAS_4_
1097	{[C-(H-Stp)_4_-H]_α,ε_-K-H-(PAS)_8_}_ε_-K	PAS_8_

**Table 2:** Particle size (Z-average), polydispersity index (PDI), and zeta potential of plasmid DNA (pDNA) polyplexes formed in HEPES-buffered 5% glucose (HBG) buffer determined with dynamic laser-light scattering (DLS). Mean of three measurements of the same sample is indicated. Polymer at nitrogen-to-phosphate ratio (N/P) 12 and 2 µg plasmid DNA (pDNA) were separately diluted with HBG pH 7.4 to 30 µL each. Then solutions were mixed and incubated for 30 min. Polyplexes were diluted to 800 µL with 10 mM NaCl pH 7.4 prior to measurement. Particle size displayed as number mean can be found in Table S2. # indicates internal library compound IDs.

Polymer #	Abbreviation	Average size (nm)	Average PDI	Average zeta potential (mV)
689	3-arm	126.8 ± 2.7	0.15 ± 0.02	32.0 ± 3.5
1088	PEG_12_	97.9 ± 1.1	0.14 ± 0.02	6.7 ± 2.3
1091	PEG_24_	111.6 ± 0.9	0.16 ± 0.01	3.1 ± 0.6
1120	PEG_48_	87.2 ± 1.4	0.33 ± 0.05	1.6 ± 0.5
1094	PAS_4_	127.7 ± 0.8	0.12 ± 0.01	7.1 ± 1.5
1097	PAS_8_	147.0 ± 1.8	0.15 ± 0.01	3.4 ± 0.8

**Table 3:** Summary of oligomer characteristics.

Experiment	Oligomers					
	3-arm	PEG_12_	PEG_24_	PEG_48_	PAS_4_	PAS_8_
PBS resistance	–	–	+	+	–	+
Erythrocyte adhesion	–	++	++	+++	+	+
Particle compaction	+++	++	+	–	++	+
Polyplex shape	globule	rod-like	doughnut aggregates	cord-like	rod-like	doughnut
Heparin resistance	+	+	–	–	+	+
Serum stability	+	+	–	–	+	–
Shielding	–	+	++	+++	+	++
Transfection (short term)	+	–	–	–	–	–
Transfection (long term)	+	+	+	v	+	+

PBS resistance: +/– indicates Yes/No; Erythrocyte adhesion: +++ lowest adhesion, ++ low adhesion, + increased adhesion, – complete adhesion; Particle compaction: +++ highest compaction, ++ mediate compaction, + compaction, – no sufficient compaction (evaluated by EtBr exclusion assay and TEM images); Heparin resistance against 250 IU: +/– indicates Yes/No; Serum stability: +/– indicates Yes/No; Shielding: +++ zeta potential (ZP) < 2 mV, ++ ZP < 5 mV, + ZP < 10 mV, – ZP > 10 mV; Transfection efficacy of polyplexes on N2a cells (short as well as long-term): + significant transfection efficacy compared to untransfected cells, – no transfection signal compared to untreated cells.

## Supplementary Materials

The following are available online at www.mdpi.com/2073-4360/9/4/142/s1; Additional methods on Figures S1–S3. Figure S1: quenching of Cy5 in pDNA polyplexes, Figure S2: Agarose gel shift (N/P 3-20), Figure S3: Agarose gel shift (N/P 0-3), Figure S4: Cell viability assay of DU145 and Huh7 after Transfection, Figure S5: Transfection of cmb-targeted oligomers on N2a cell line, Figure S6: Dde-K-(SAP)_8_ MALDI-MS, Figure S7: ^1^H-NMR spectrum of #689, Figure S8: RP-HPLC spectrum of #689, Figure S9: ^1^H-NMR spectrum of #1088, Figure S10: RP-HPLC spectrum of #1088, Figure S11: ^1^H-NMR spectrum of #1091, Figure S12 RP-HPLC spectrum of #1091, Figure S13: ^1^H-NMR spectrum of #1120, Figure S14: RP-HPLC spectrum of #1120, Figure S15: ^1^H-NMR spectrum of #1094, Figure S16: RP-HPLC spectrum of #1094, Figure S17: ^1^H-NMR spectrum of #1097, Figure S18: RP-HPLC spectrum of #1097, Figure S19: ^1^H-NMR spectrum of #1078, Figure S20: RP-HPLC spectrum of #1078, Figure S21: ^1^H-NMR spectrum of #996, Figure S22: RP-HPLC spectrum of #996, Figure S23: ^1^H-NMR spectrum of #442, Figure S24: RP-HPLC spectrum of #442, Figure S25: ^1^H-NMR spectrum of #694, Figure S26: RP-HPLC spectrum of #694, Figure S27: ^1^H-NMR spectrum of #1000, Figure S28: RP-HPLC spectrum of #1000, Figure S29: ^1^H-NMR spectrum of #901, Figure S30: RP-HPLC spectrum of #901, Table S1: Average sizes of oligomers (Number mean), Table S2: List of c-Met targeted oligomers.
