# Supplementary material for: Influence of Defined Hydrophilic Blocks within Oligoaminoamide Copolymers: Compaction versus Shielding of pDNA Nanoparticles
Source: Polymers (Basel). 2017 Apr 19;9(4):142. doi: 10.3390/polym9040142 (PMC6432433; doi:10.3390/polym9040142)
Supplement: Supplementary file 1 [file polymers-09-00142-s001.pdf]

Supplementary Materials: Influence of Defined Hydrophilic Blocks within Oligoaminoamide Copolymers: Compaction versus Shielding of pDNA Nanoparticles

Stephan Morys, Ana Krhac Levacic, Sarah Urnauer, Susanne Kempter, Sarah Kern, Joachim Rädler, Christine Spitzweg, Ulrich Lächelt and Ernst Wagner

Loading of a Chlorotrityl Chloride Resin with Fmoc-Cys(Trt)-OH

Chlorotrityl chloride resin (1.2 mmol chloride, 750 mg) was swollen in 7 mL dry DCM for 10 min, a solution of 527.14 mg Fmoc-Cys(Trt)-OH (0.9 mmol, 0.75 eq.) in 5 mL DCM and 470.3 µL DIPEA (2.7 mmol, 2.25 eq.) were added and the resin was shaken for 1 h in an overhead shaker. The reaction solvent was drained and 5 mL of a mixture of dry DCM/MeOH/DIPEA (80/15/5) was added for 30 min. After the removal of the reaction mixture, the resin was washed 5 times with DCM.

Forty mg of the wet resin were separated and dried to determine the loading of the resin. Therefore, an exactly weighted amount of the dry resin was treated with 1 mL deprotection solution (20% piperidine in DMF) for 1 h. Afterwards, the solution was diluted and absorption was measured at 301 nm. The loading was then calculated according to the equation: resin load [mmol/g] = (A × 1000)/(m [mg] × 7800 × d_f) with d_f as dilution factor.

The resin was treated five times with 20% piperidine in DMF to remove the fmoc protection group. Successful deprotection was evaluated by Kaiser test [1]. Afterwards, the resin was washed with DMF, DCM and n-hexane and dried in vacuo.

Loading of a Chlorotrityl Chloride Resin with Dde-Lys(Fmoc)-OH

The loading was performed analogously to the loading of a chlorotrityl chloride resin with Fmoc-Cys(Trt)-OH. Instead of Fmoc-Cys(Trt)-OH, Dde-Lys(Fmoc)-OH (479.4 mg) was used as amino acid.

Loading of a Chlorotrityl Chloride Resin with Fmoc-Lys(Dde)-OH

The loading was performed similar to the loading of a chlorotrityl chloride resin with Dde-Lys(Fmoc)-OH. Instead of Dde-Lys(Fmoc)-OH, Fmoc-Lys(Dde)-OH (479.4 mg) was used as amino acid.

Synthesis of cmb Containing Oligomers

For hepatocyte growth factor (HGF) receptor/cMet targeted delivery, oligomers containing a c-Metet binding peptide (cmb) were synthesized. Chlorotrityl chloride resin preloaded with Fmoc-Lys(Dde)-OH was used. After Fmoc removal with 5 times 20% piperidine, and a Kaisers Test for verification of the deprotection, the cmb ligand (KSLSRHDHIHHH) was synthesized with a Syro Wave (Biotage, Uppsala, Sweden), following the synthesis protocol as previously described in the main manuscript. The terminal lysine was bocylated to terminate synthesis at this arm of the lysine. Then Dde deprotection at the side chain was conducted with

15 cycles of 4% hydrazine in DMF (*v*/*v*) for 3 min each. Afterwards the resin was split into 6 aliquots. For the cmb targeted oligomers PEG_{12}, PEG_{24}, and PEG_{48} either Fmoc-N-amido-$dPEG_{12}$-OH or Fmoc-N-amido-$dPEG_{24}$-OH was coupled as described in the main manuscript. PAS_4 or PAS_8 was synthesized by successive coupling of (Fmoc-L-Ser(tBu)-OH, Fmoc-L-Ala-OH and Fmoc-L-Pro-OH) repetitively four or eight times, again with the Syro Wave, following the coupling and deprotection protocol of the main manuscript. Afterwards the cationic backbone again was built with Fmoc-L-His(Trt)-OH, Fmoc-L-Lys(Fmoc)-OH, Fmoc-Stp$(Boc)_3$-OH and terminated with double couplings at room temperature of Boc-L-Cys(Trt)-OH for 1 h each, this again was performed in accordance with the protocol mentioned in the main manuscript. Instead of a hydrophilic shielding block, cmb-3-arm was built of three cationic arms containing with alternating histidine and Stp. Synthesis followed the same coupling procedure as of the 3-arm, with the only difference that the C-terminal arm was not started with a Fmoc-L-Cys(Trt)-OH, but was attached to the H_2N-cmb-K, previously synthesized. A detailed step by step description of the synthesis of cmb-PEG_{24} can be found in [2], being applicable to all 2-arm structures after synthesis of the shielding block. After the final coupling step, the resins were dried and the products cleaved off the resin for 90 min using a mixture of TFA/EDT /H_2O/TIS 94:2.5:2.5:1 (*v*/*v*) at a ratio of 10 mL per gram resin. The cleaved oligomers were purified by size exclusion chromatography (SEC) performed with 10 mM hydrochloric acid / acetonitrile 7:3 as solvent. An ÄKTA purifier system (GE Healthcare Biosciences, Uppsala, Sweden) equipped with a Sephadex G-10 column and a P-900 solvent pump module, a UV-900 spectrophotometrical detector, a pH/C-900 conductivity module, a Frac-950 automated fractionator was used. The product fractions were collected and combined prior to lyophilization.

Analytical data of ^{1}H-NMR and RP-HPLC can be found below.

Oligomer sequences of all cmb containing structures can be found from N to C terminus in Table S1.

pDNA Binding Assay by Electrophoresis

For investigation of the pDNA binding ability of the oligomers, a 1% agarose gel was prepared. Therefore, agarose was suspended in TBE buffer (trizma base 10.8 g, boric acid 5.5 g, EDTA 0.75 g per 1 L of of water) and the mixture was heated until boiling and total dissolution. After cooling down to approximately 60 °C, GelRed (Biotium Inc., Hayward, CA, USA) for detection of the nucleic acid was added and the agarose solution was casted into an electrophoresis unit and left to form a solid gel. Then polyplexes consisting of oligomers at N/P 3, 6, 12 and 20 were prepared with 200 ng pDNA in a total volume of 20 µL. After 30 min, 4 µL of 6× loading buffer (6 mL of glycerol, 1.2 mL of 0.5 M EDTA, 2.8 mL of H_2O, 0.02 g of bromophenol blue) was added to each sample and applied to the gel. Electrophoresis was performed for 80 min at 120 V.

pDNA Compaction Assay/Cy-5 Quenching Assay

Polyplexes of indicated oligomers were formed with 2 µg pDNA (80% pCMV-Luc, 20% Cy5 labeled pCMV-Luc) at N/P 12 in a total volume of 60 µL. Then either 1400 µL of Hepes buffered glucose (HBG pH = 7.4) or 700 µL HBG and 700 µL heparin (containing 3500 IU) were added and gently mixed. Cy5 excitation/emission was measured at 649/670 nm with a Cary Eclipse spectrophotometer (Varian, Germany). Results are calculated against HBG as blank and free pDNA represents 100%.

Maldi Mass Spectrometry

One µL matrix consisting of a saturated solution of Super-DHB (mixture of 2,5-Dihydroxybenzoic acid and 2-hydroxy-5-methoxybenzoic acid) in acetonitrile / water (1:1) containing 0.1% (*v*/*v*) trifluoroacetic acid was applied on a MTP AnchorChip (Bruker Daltonics, Bremen, Germany). After the Super-DHB matrix dried and crystalized, one µL of the sample solution (10 mg/mL in water) was added to the matrix spot. Samples were analyzed using an Autoflex II mass spectrometer (Bruker Daltonics, Bremen, Germany).

Analytical RP-HPLC

Reversed-phase HPLC (RP-HPLC) was carried out with a VWR-Hitachi Chromaster 5160 Pump System (VWR, Darmstadt, Germany), VWR-Hitachi Chromaster 5260 Autosampler (VWR, Darmstadt, Germany) and a Diode Array Detector (VWR-Hitachi Chromaster 5430; VWR, Darmstadt, Germany) at 214 nm detection wavelength. As a column either a YMC Hydrosphere 302 C_{18} (YMC Europe, Dinslaken, Germany) or a Waters Sunfire C_{18} (Waters, Saint-Quentin En Yvelines Cedex, France) was used. A gradient starting at 95:5 (water:acetonitrile) to 0:100 within 20 min was applied. All solvents were supplemented with 0.1% trifluoroacetic acid.

Proton NMR Spectroscopy

^{1}H NMR spectra were recorded using an AVANCE III HD500 (500 MHz) by Bruker with a 5 mm CPPBBO probe (Bruker Daltonics, Bremen, Germany). Spectra were recorded without TMS as internal standard and therefore all signals were calibrated to the residual proton signal of the deuterium oxide (D_2O) solvent. Chemical shifts are reported in ppm and refer to the solvent as internal standard (D_2O at 4.79). Integration was performed manually. The spectra were analyzed using MestreNova (Ver.9.0 by MestReLab Research). Integrals were normalized to the succinic acid peaks.

Table S1. Average sizes of oligomers displayed as number mean in nm. # indicates internal library compound IDs.

Polymer	Abbreviation	Average size
689	3-arm	83.4 ± 0.7
1088	PEG_{12}	67.9 ± 0.6
1091	PEG_{24}	76.6 ± 3.5
1120	PEG_{48}	38.8 ± 5.7
1094	PAS_4	87.0 ± 3.5
1097	PAS_8	91.3 ± 12.3

Table S2. List of c-Met targeted oligomers. cmb represents c-Met binding peptide, structures from N to C terminus, as well as an abbreviation used within the paper. # indicates internal library compound IDs.

Polymer #	Structure	Abbreviation
1078	cmb-[(C-(H-Stp)$_3$-H)$_{\alpha,\varepsilon}$K-H-(Stp-H)$_3$]$_\varepsilon$K	cmb-3-arm
996	cmb-[(C-(H-Stp)$_4$-H)$_{\alpha,\varepsilon}$K-H-dPEG$_{12}$]$_\varepsilon$K	cmb-PEG$_{12}$
442	cmb-[(C-(H-Stp)$_4$-H)$_{\alpha,\varepsilon}$K-H-dPEG$_{24}$]$_\varepsilon$K	cmb-PEG$_{24}$
694	cmb-[(C-(H-Stp)$_4$-H)$_{\alpha,\varepsilon}$K-H-dPEG$_{24}$-dPEG$_{24}$]$_\varepsilon$K	cmb-PEG$_{48}$
1000	cmb-[(C-(H-Stp)$_4$-H)$_{\alpha,\varepsilon}$K-H-(PAS)$_4$]$_\varepsilon$K	cmb-PAS$_4$
901	cmb-[(C-(H-Stp)$_4$-H)$_{\alpha,\varepsilon}$K-H-(PAS)$_8$]$_\varepsilon$K	cmb-PAS$_8$

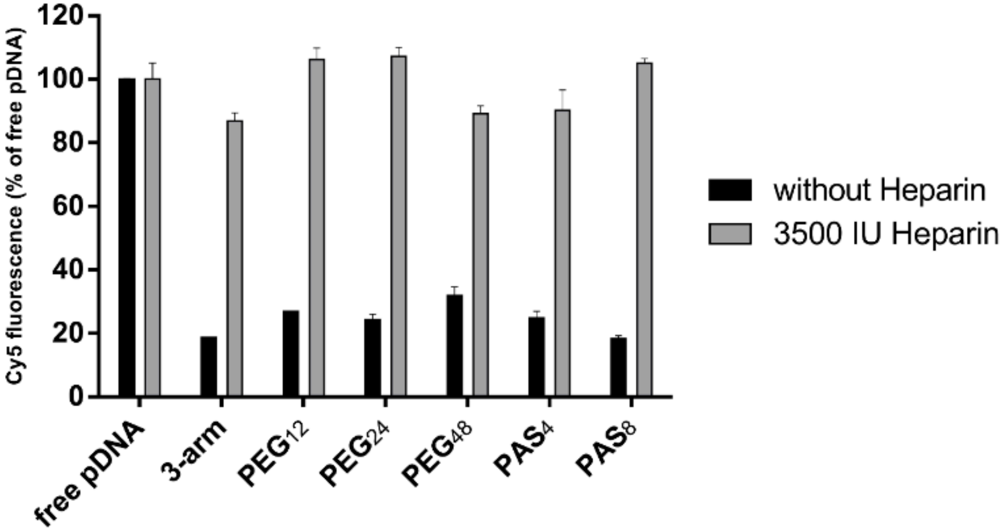

Figure S1. Self-quenching of Cy5 labeled pDNA (black bars) and release from polyplexes after heparin addition (grey bars). Uncomplexed pDNA served as control. Results are displayed as percent of control.

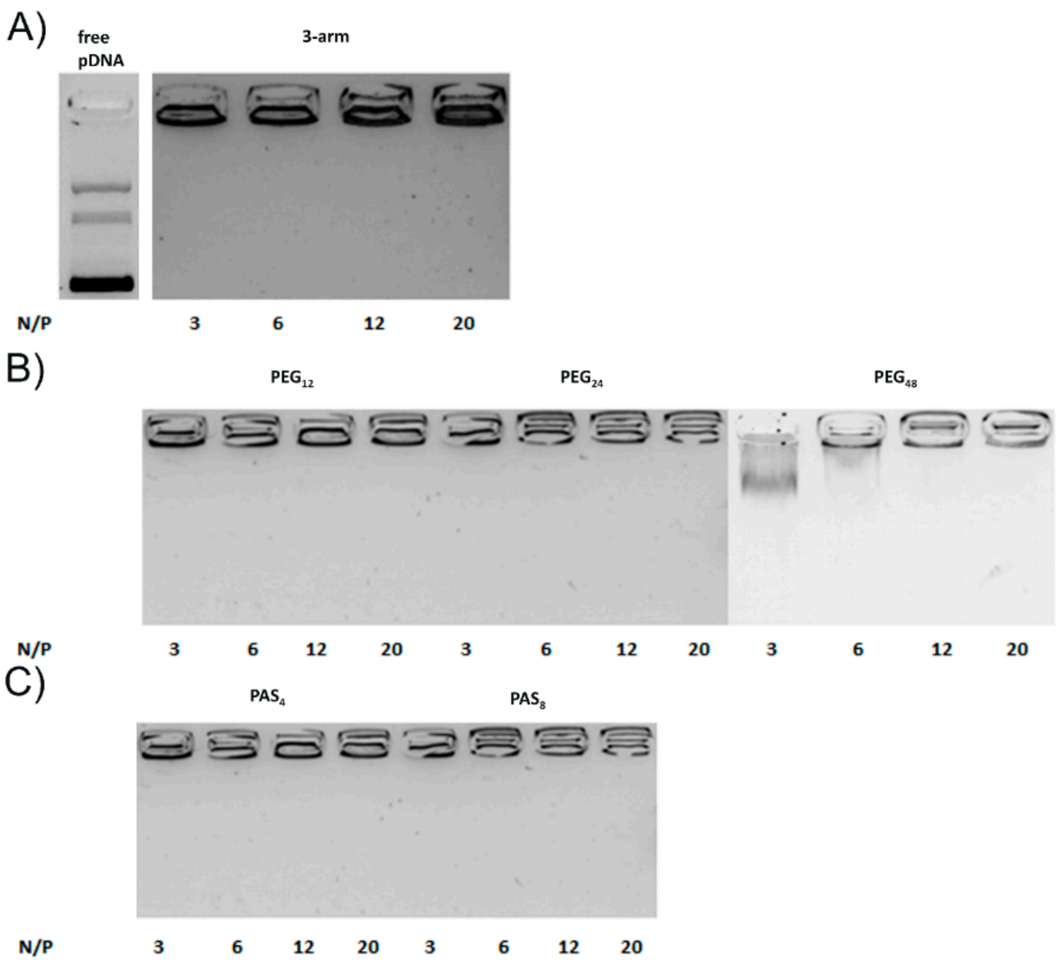

Figure S2. Retardation of formed pDNA complexes in agarose gel at indicated N/P with non shielded 3-arm (**A**) PEGylated (**B**) and PASylated (**C**) oligomers. Left lane: free pDNA.

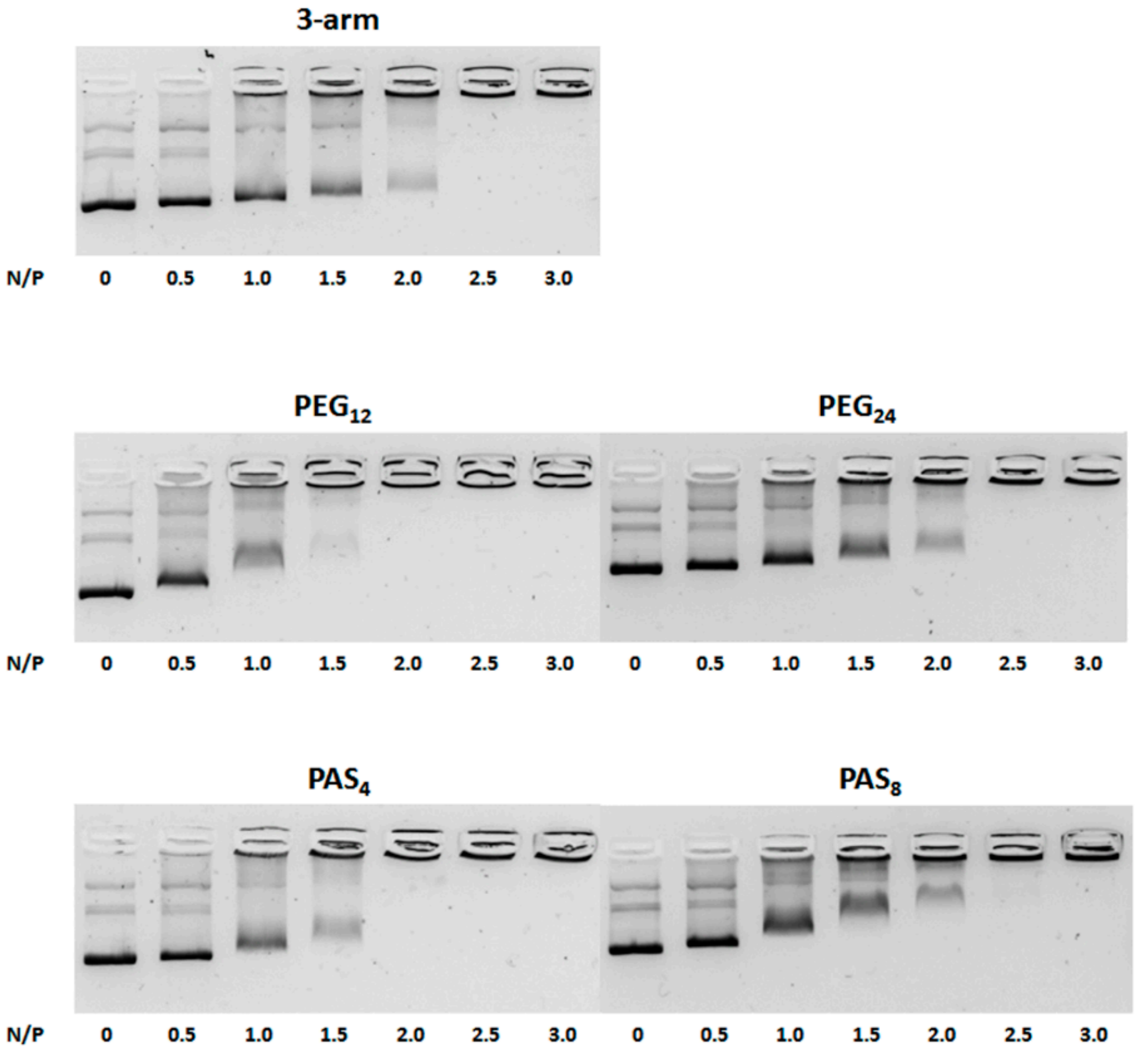

Figure S3. Titration of N/P of untargeted polyplexes between N/P of 0 (free pDNA) and 3 in an agarose gel shift assay.

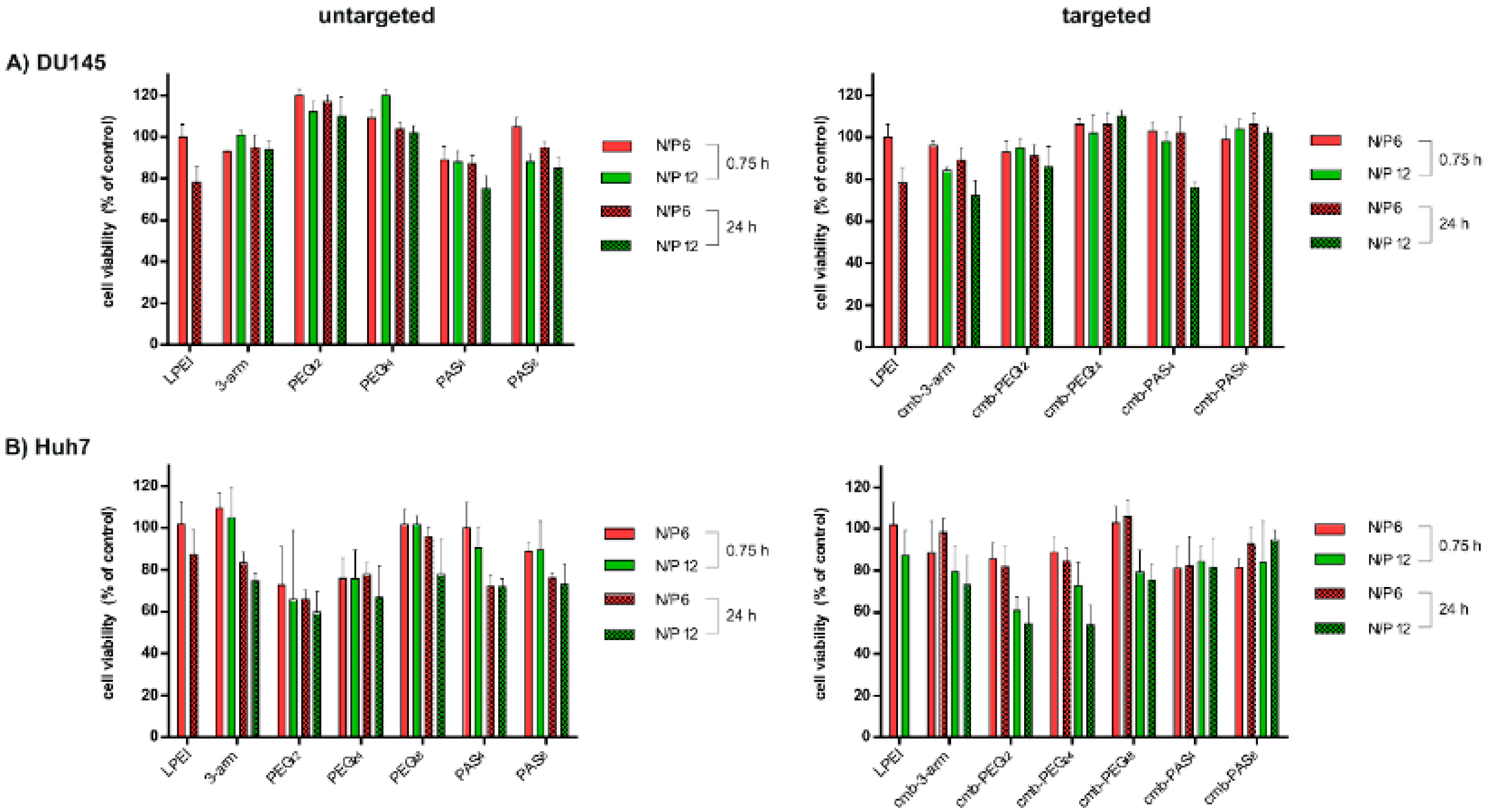

Figure S4. Cell viability assay of (**A**) DU145 and (**B**) Huh7 was performed in parallel to luciferase transfection. An MTT assay was performed after 0.75 h (without pattern), and after 24h (with pattern) at two different N/P ratios, N/P = 6 (red) and N/P = 12 (green).

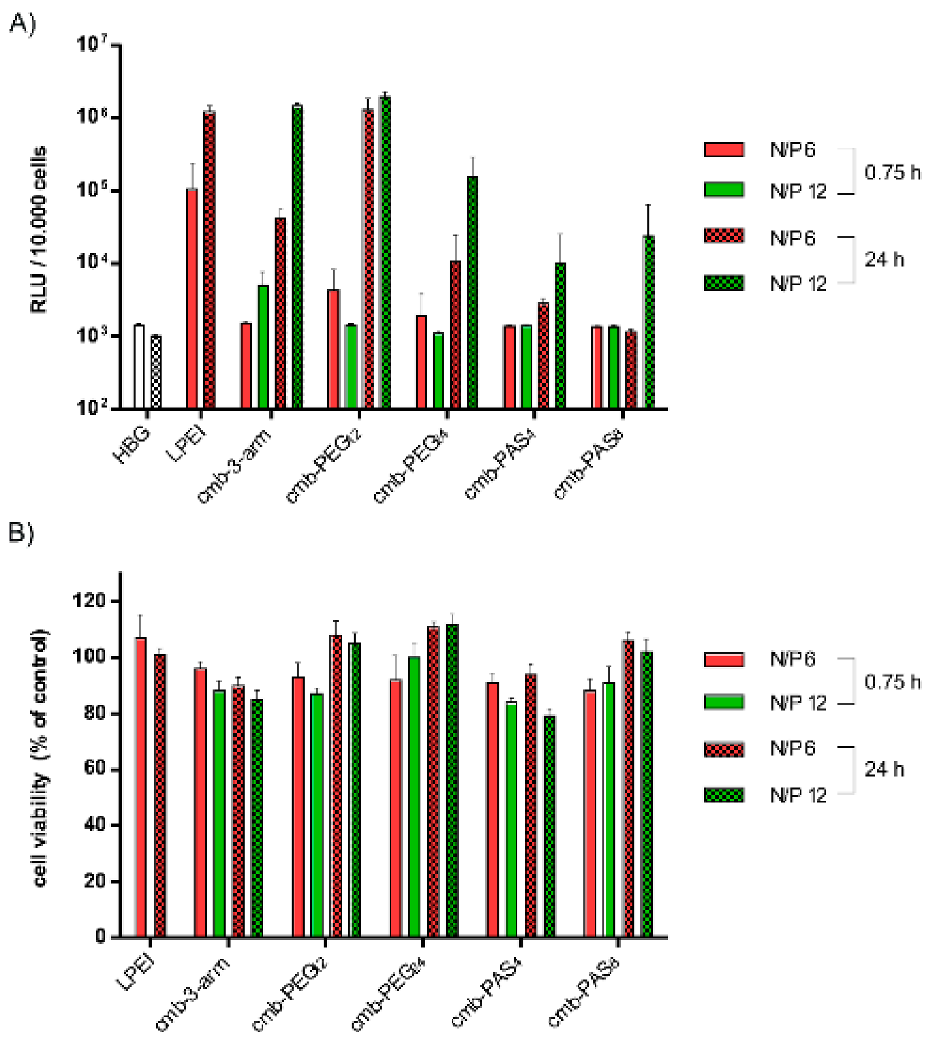

Figure S5. (**A**) Luciferase reporter gene expression in c-Met negative cell line N2a after 0.75 h (without pattern) and after 24 h (with pattern). Transfections were performed at two different N/P rations, N/P = 6 (red) and N/P = 12 (green). (**B**) An MTT assay was performed after 0.75 h (without pattern) in parallel after 24 h (with pattern) at two different N/P ratios, N/P = 6 (red) and N/P = 12 (green).

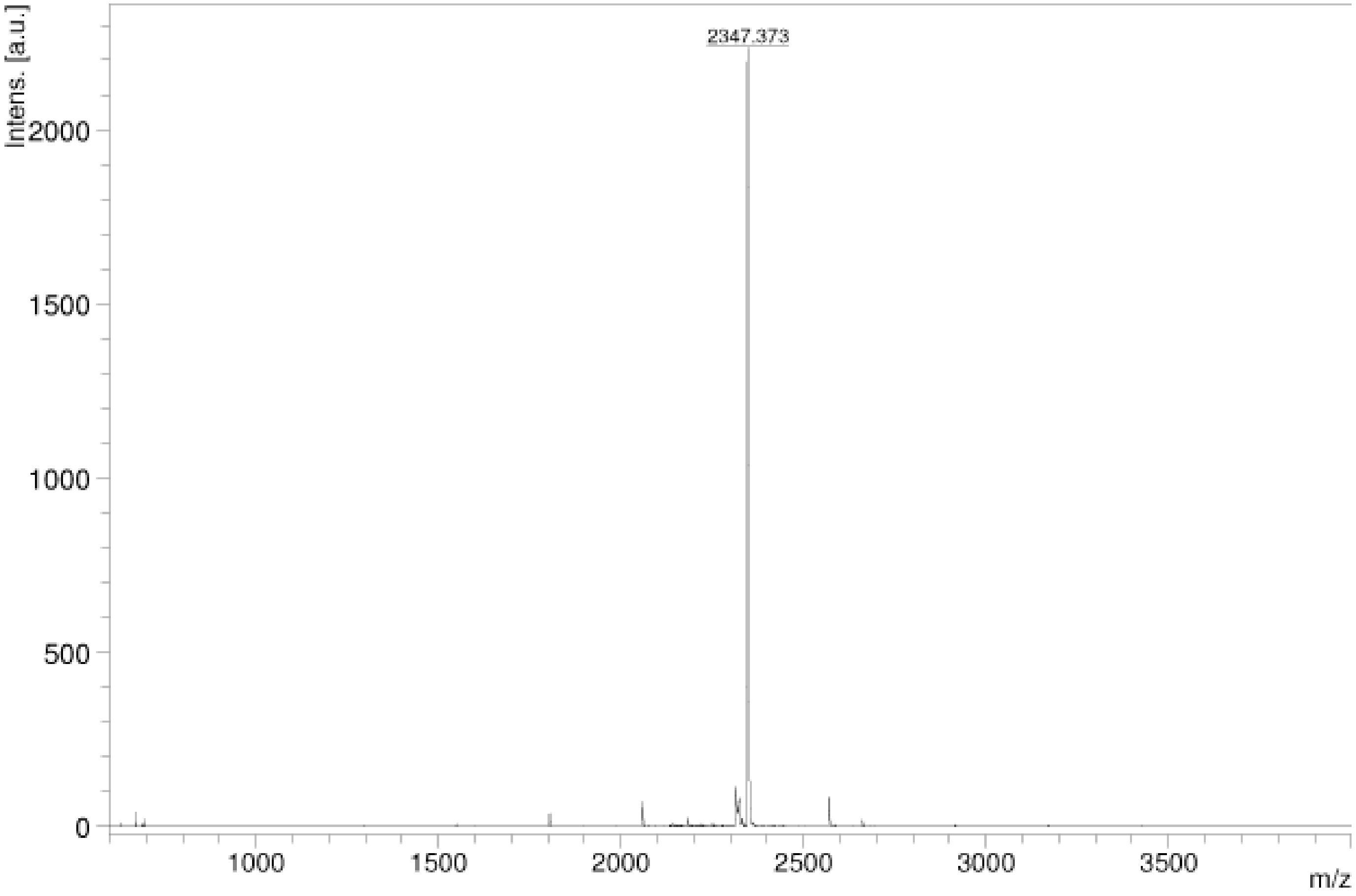

Figure S6. Dde-K-(SAP)$_8$ MALDI-MS: Calculated mass [M–H] of $C_{104}H_{161}N_{26}O_{36}$: 2350.16 g/mol.

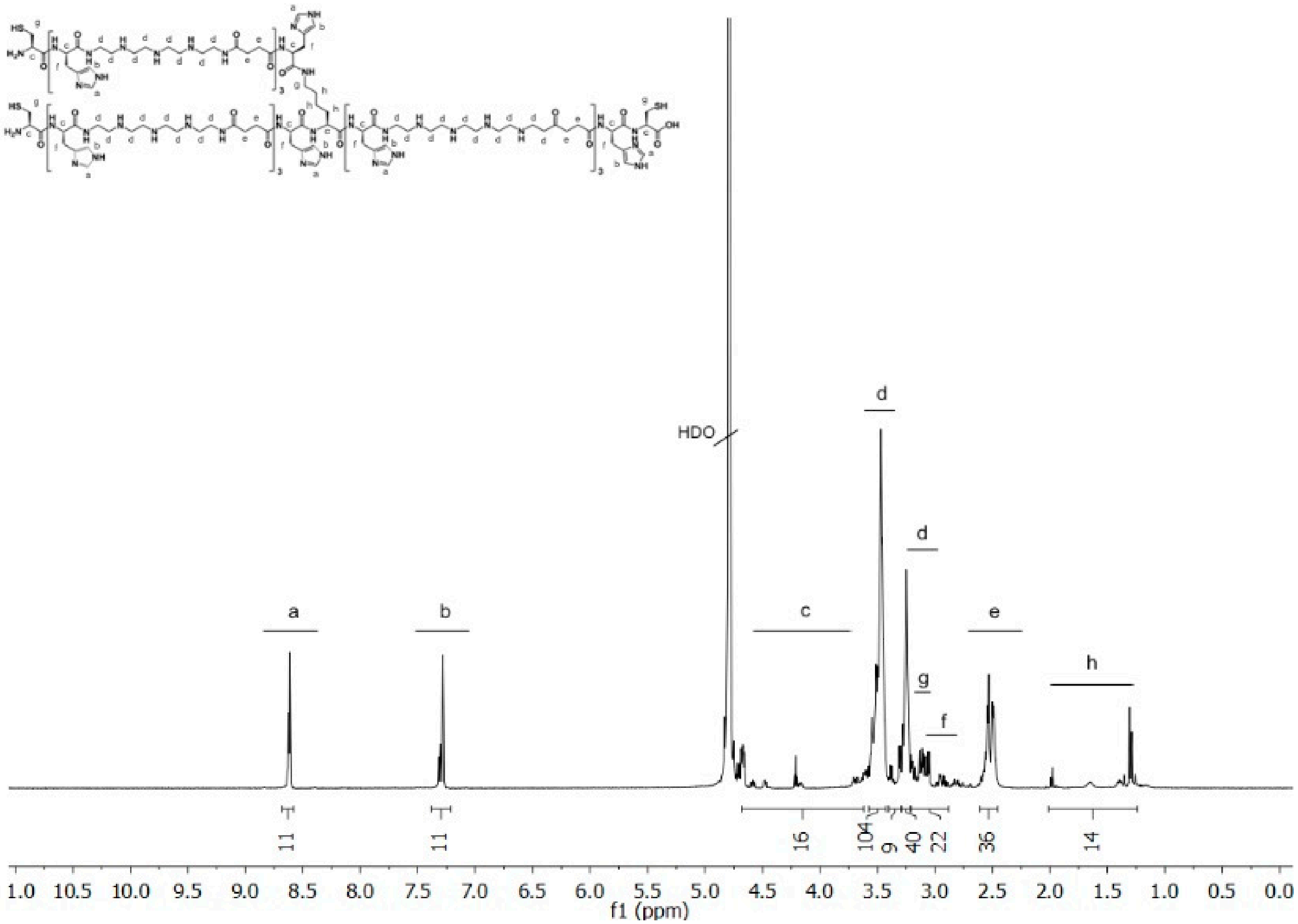

Figure S7. ^{1}H-NMR spectrum of 3-arm (#689) in D_2O. Sequence (N–>C): [C–(H–Stp)$_3$–H]$_{\alpha,\varepsilon}$–K–H–(Stp-H)$_3$–C δ (ppm) = 1.1–1.4 (comp, 6H, βγδH lysine), 2.3–2.7 (comp, 36 H, –CO–CH_2–CH_2–CO– succinic acid), 2.9-3.8 (comp, 176 H, –CH_2– tepa, βH cysteine, βH histidine, εH lysine), 4.1–4.7 (comp, 16 H, αH cysteine, lysine, histidine), 4.79 (s, HDO), 7.2–7.4 (m, 11 H, aromatic H histidine), 8.5–8.7 (m, 11 H, aromatic H histidine). comp indicates a group of overlaid protons.

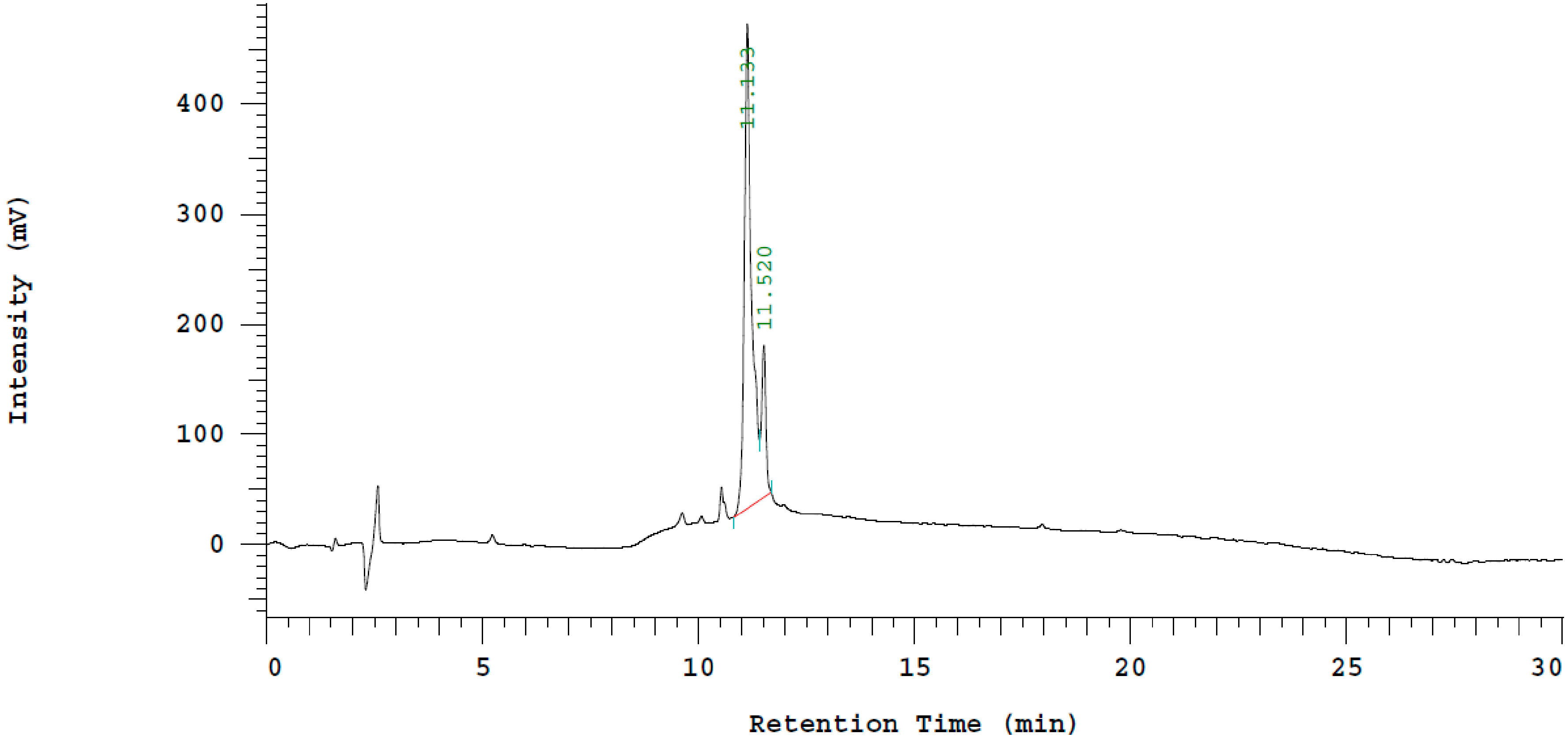

Figure S8. RP-HPLC of 3-arm (#689).

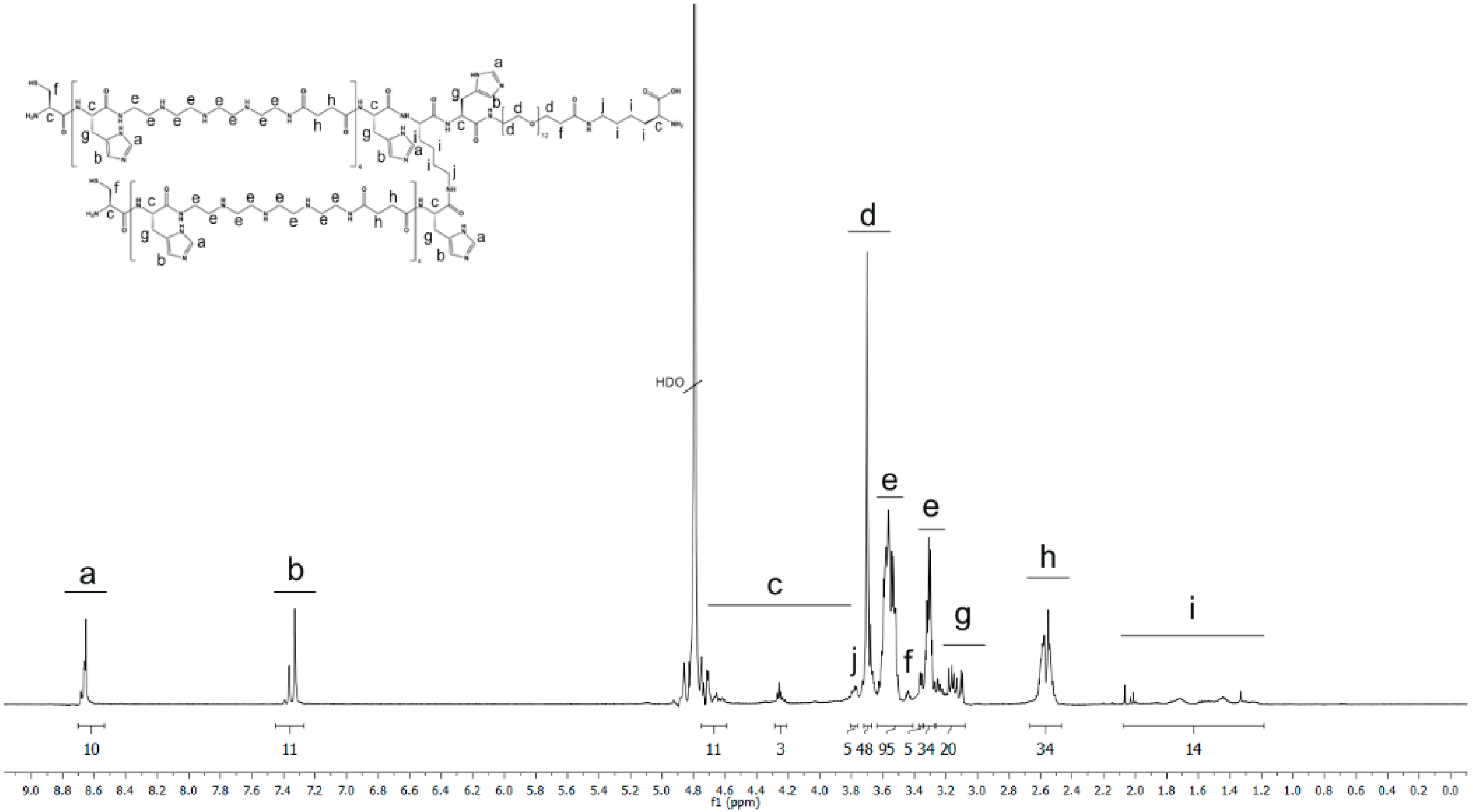

Figure S9. ^{1}H-NMR spectrum of PEG_{12} (#1088) in D_2O. Sequence (N–>C): {[C–(H-Stp)$_4$–H]$_{\alpha,\varepsilon}$–K–H–dPEG$_{12}$}$_\varepsilon$–K. δ (ppm) = 1.1–1.4 (comp, 12H, βγδH lysine), 2.3–2.7 (comp, 34 H, –CO–CH_2–CH_2–CO– succinic acid –CO–CH2–dPEG$_{12}$), 2.9-3.65 (comp, 154 H, –CH_2– tepa, βH cysteine, βH histidine), 3.70 (s, 48H, –CH2–O–dPEG$_{12}$, –CH2–N–dPEG$_{12}$), 3.75–3.85 (m, 4H, εH lysine) 4.1–4.7 (comp, 15 H, αH cysteine, lysine, histidine), 4.79 (s, HDO), 7.2–7.4 (m, 11 H, aromatic H histidine), 8.5–8.7 (m, 11 H, aromatic H histidine). comp indicates a group of overlaid protons.

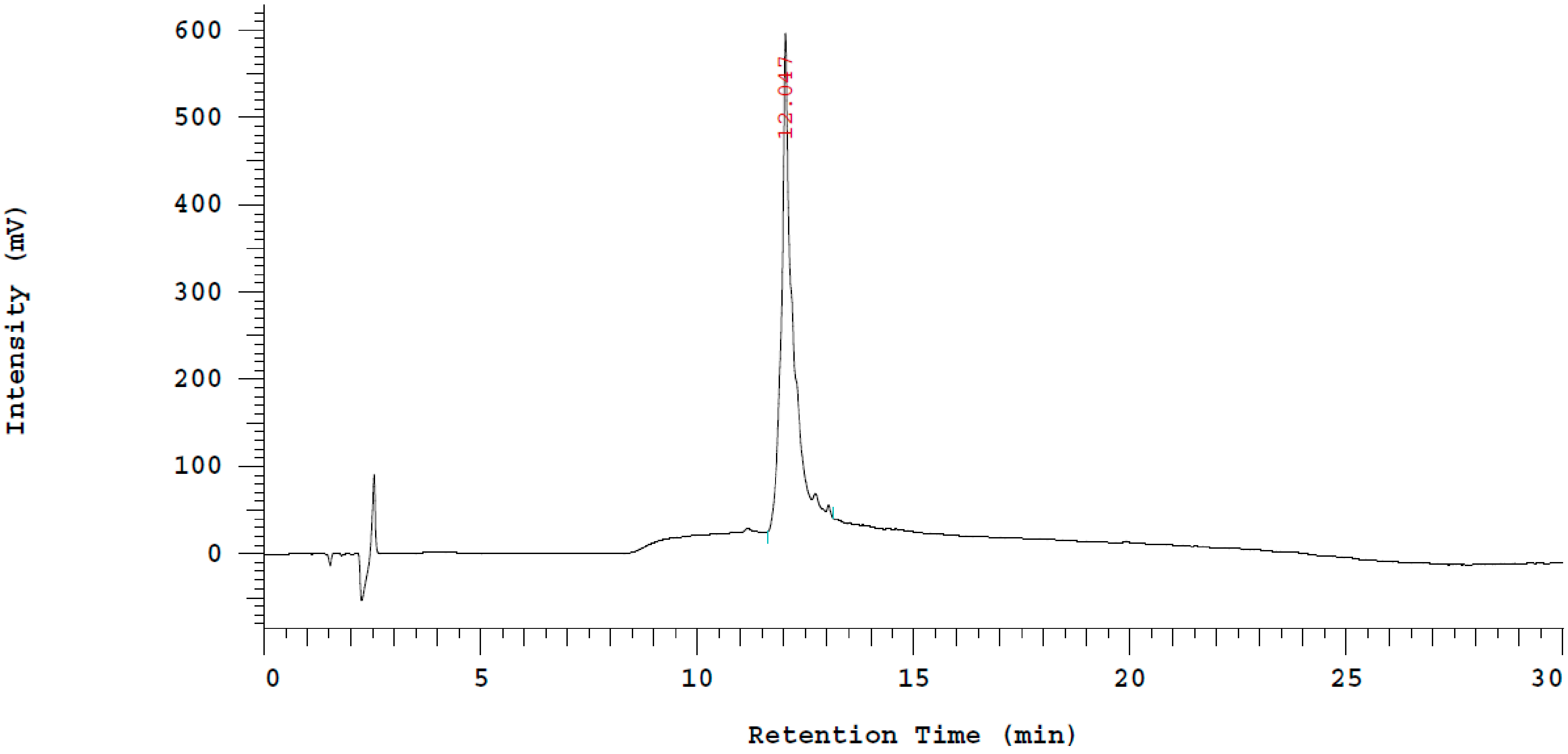

Figure S10. RP-HPLC of PEG_{12} (*#1088*).

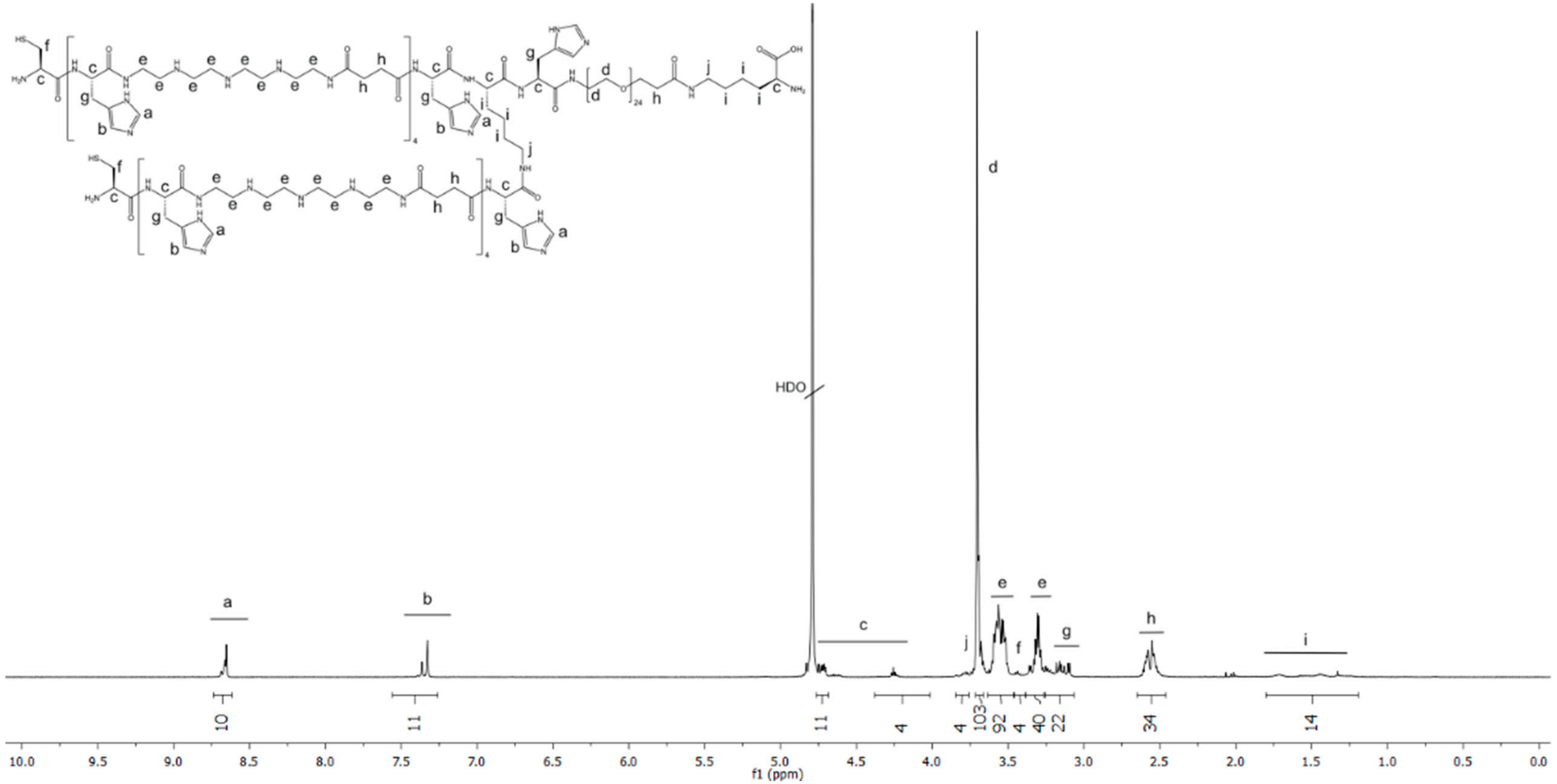

Figure S11. ^{1}H-NMR spectrum of PEG_{24} (#1091) in D_2O. Sequence (N->C): {[C-(H-Stp)$_4$-H]$_{\alpha,\varepsilon}$-K-H-dPEG$_{24}$}$_\varepsilon$-K. δ (ppm) = 1.1–1.4 (comp, 12H, βγδH lysine), 2.3–2.7 (comp, 34 H, –CO–CH_2–CH_2–CO– succinic acid –CO–CH2–dPEG$_{24}$), 2.9-3.65 (comp, 154 H, –CH_2– tepa, βH cysteine, βH histidine), 3.70 (s, 98H, –CH2–O–dPEG$_{24}$, –CH2–N–dPEG$_{24}$), 3.75–3.85 (m, 4H, εH lysine) 4.1–4.7 (comp, 15 H, αH cysteine, lysine, histidine), 4.79 (s, HDO), 7.2–7.4 (m, 11 H, aromatic H histidine), 8.5–8.7 (m, 11 H, aromatic H histidine). comp indicates a group of overlaid protons.

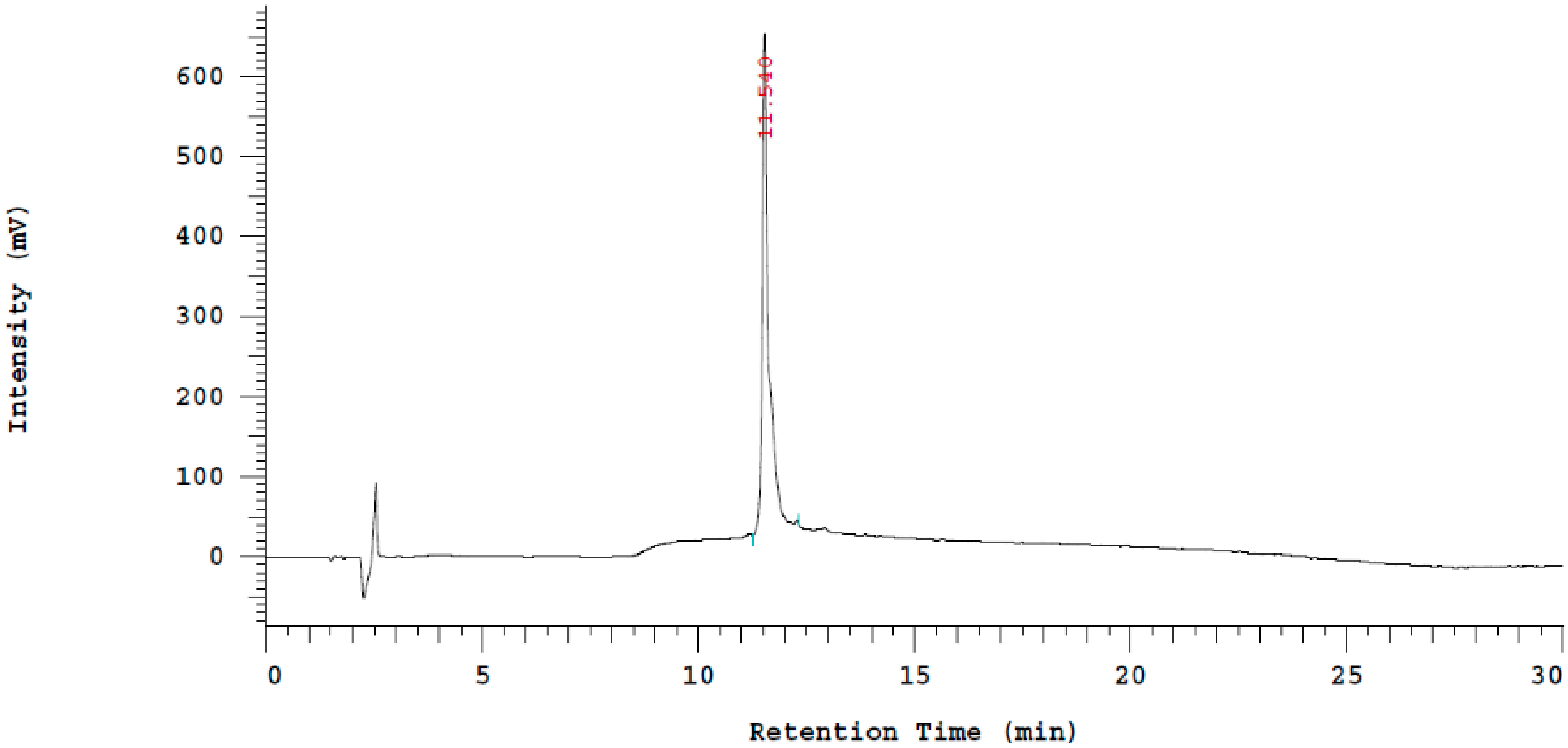

Figure S12. RP-HPLC of PEG_{24} (#1091).

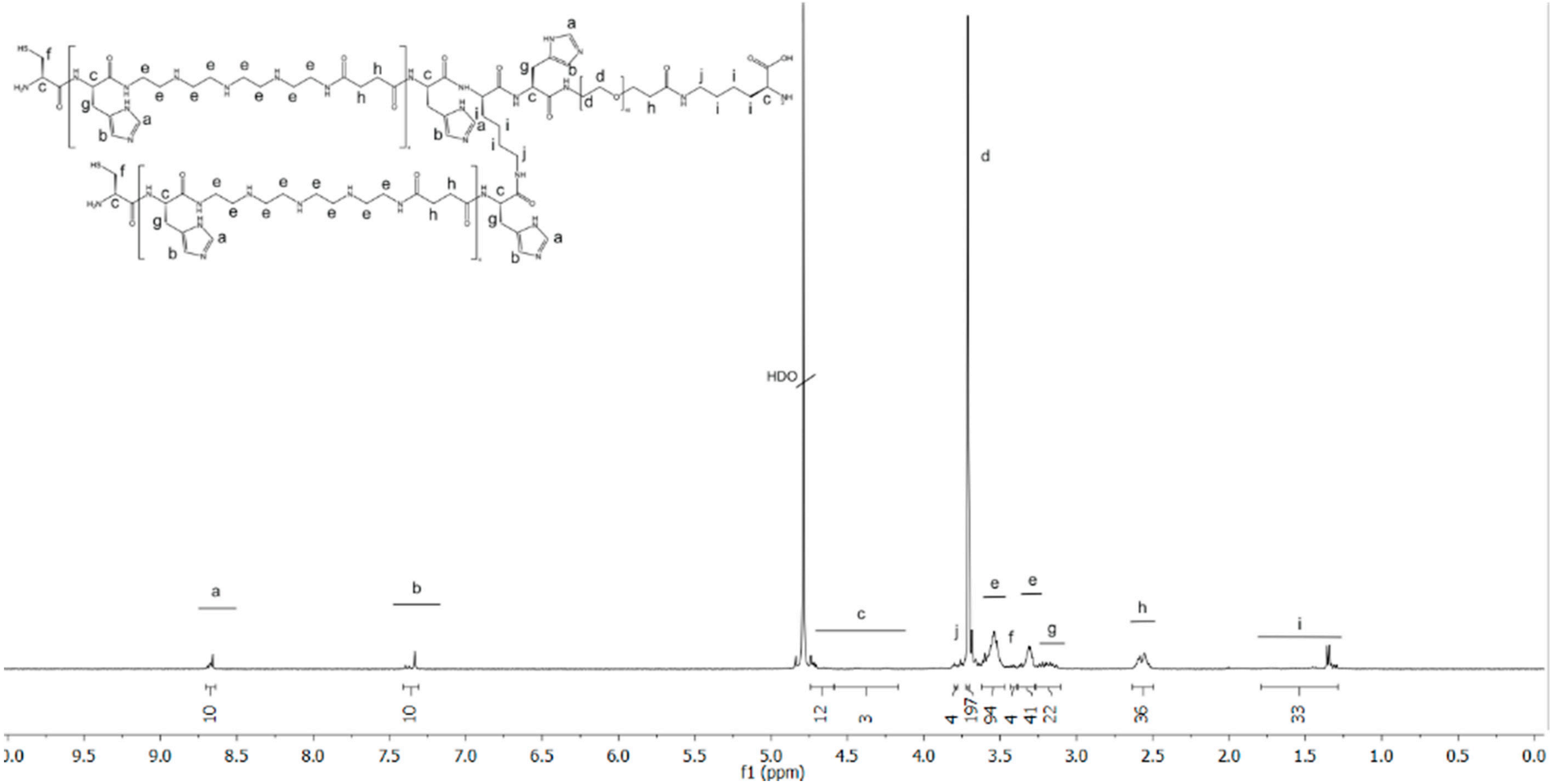

Figure S13. ^{1}H-NMR spectrum of PEG_{48} (#1120) in D_2O. Sequence (N->C): {[C-(H-Stp)$_4$-H]$_{\alpha,\varepsilon}$-K-H-dPEG$_{24}$-dPEG$_{24}$}$_\varepsilon$-K.δ (ppm) = 1.1–1.4 (comp, 12H, βγδH lysine), 2.3–2.7 (comp, 34 H, –CO–CH_2–CH_2–CO– succinic acid –CO–CH2–dPEG$_{24}$), 2.9-3.65 (comp, 154 H, –CH_2– tepa, βH cysteine, βH histidine), 3.70 (s, 196H, –CH2–O–dPEG$_{24}$, –CH2–N–dPEG$_{24}$), 3.75-3.85 (m, 4H, εH lysine) 4.1–4.7 (comp, 15 H, αH cysteine, lysine, histidine), 4.79 (s, HDO), 7.2–7.4 (m, 11 H, aromatic H histidine), 8.5–8.7 (m, 11 H, aromatic H histidine). comp indicates a group of overlaid protons.

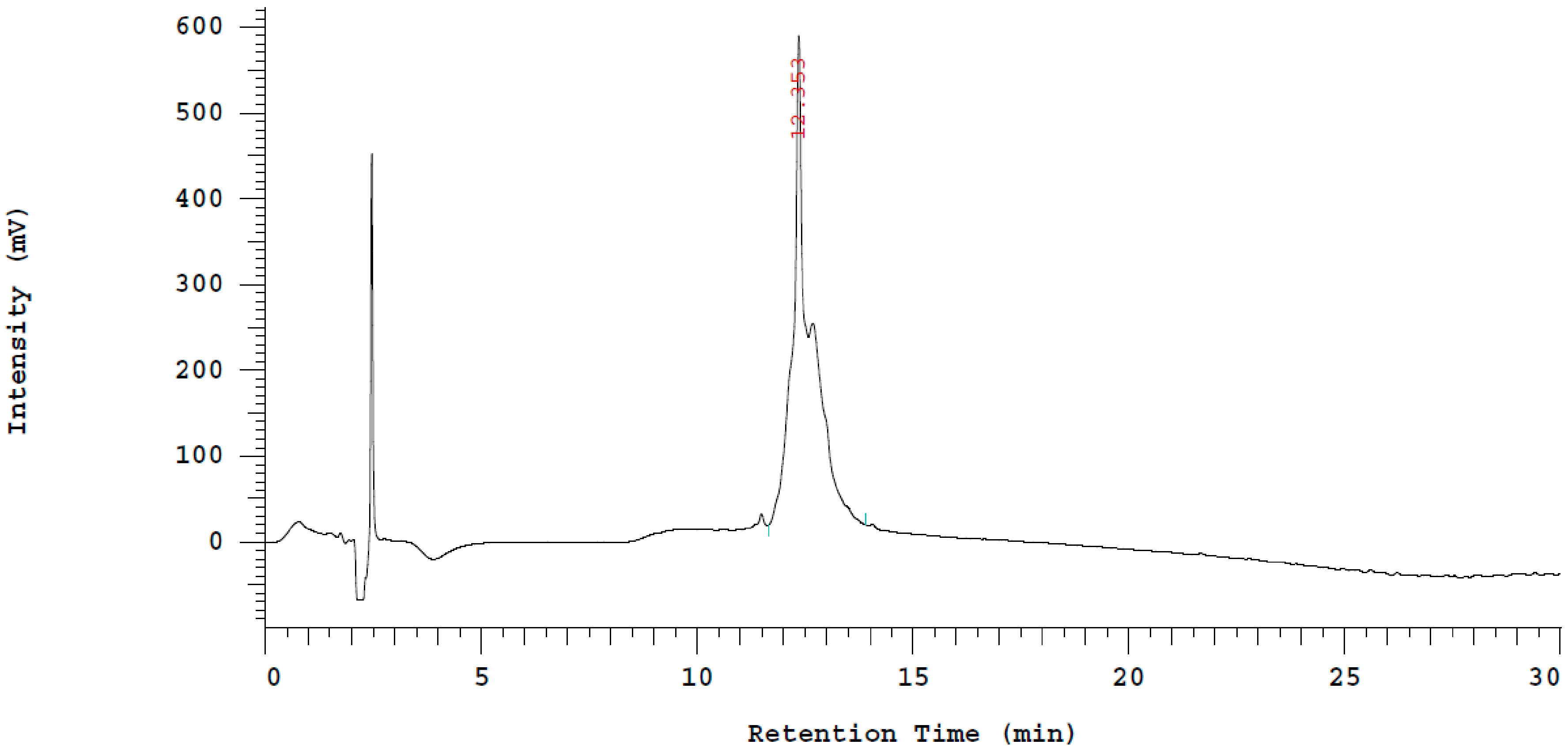

Figure S14. RP-HPLC of PEG_{48} (#1120).

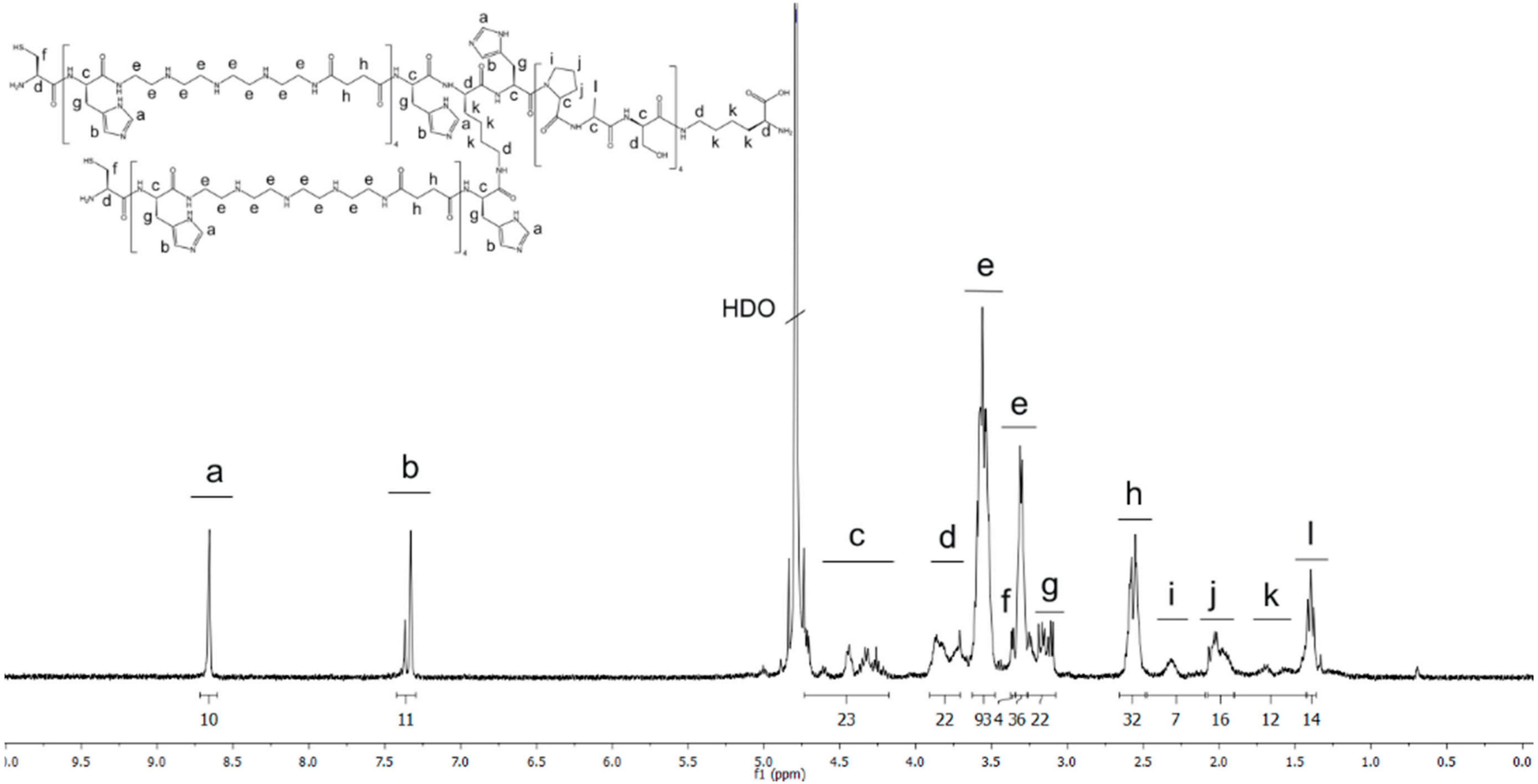

Figure S15. ^{1}H-NMR spectrum of PAS_4 (#1094) in D_2O. Sequence (N–>C): {[C–(H–Stp)$_4$–H]$_{\alpha,\varepsilon}$–K–H–(PAS)$_4$}$_\varepsilon$–K.δ (ppm) = 1.3–1.4 (td, 12H, –CH_3–H alanine), 1.45–1.9 (comp, 12H βγδH lysine), 1.95–2.45 (comp, 24H, –CH2 proline), 2.5–2.7 (comp, 32 H, –CO–CH_2–CH_2–CO– succinic acid), 3.1–3.65 (comp, 154 H, –CH_2– tepa, βH cysteine, βH histidine), 3.70–3.90 (m, 16H, αεH lysine, βH serine, αH cysteine) 4.1–4.7 (comp, 23 H, αH histidine, αH serine, αH proline, αH alanine), 4.79 (s, HDO), 7.2–7.4 (m, 11 H, aromatic H histidine), 8.5–8.7 (m, 11 H, aromatic H histidine). Comp indicates a group of overlaid protons.

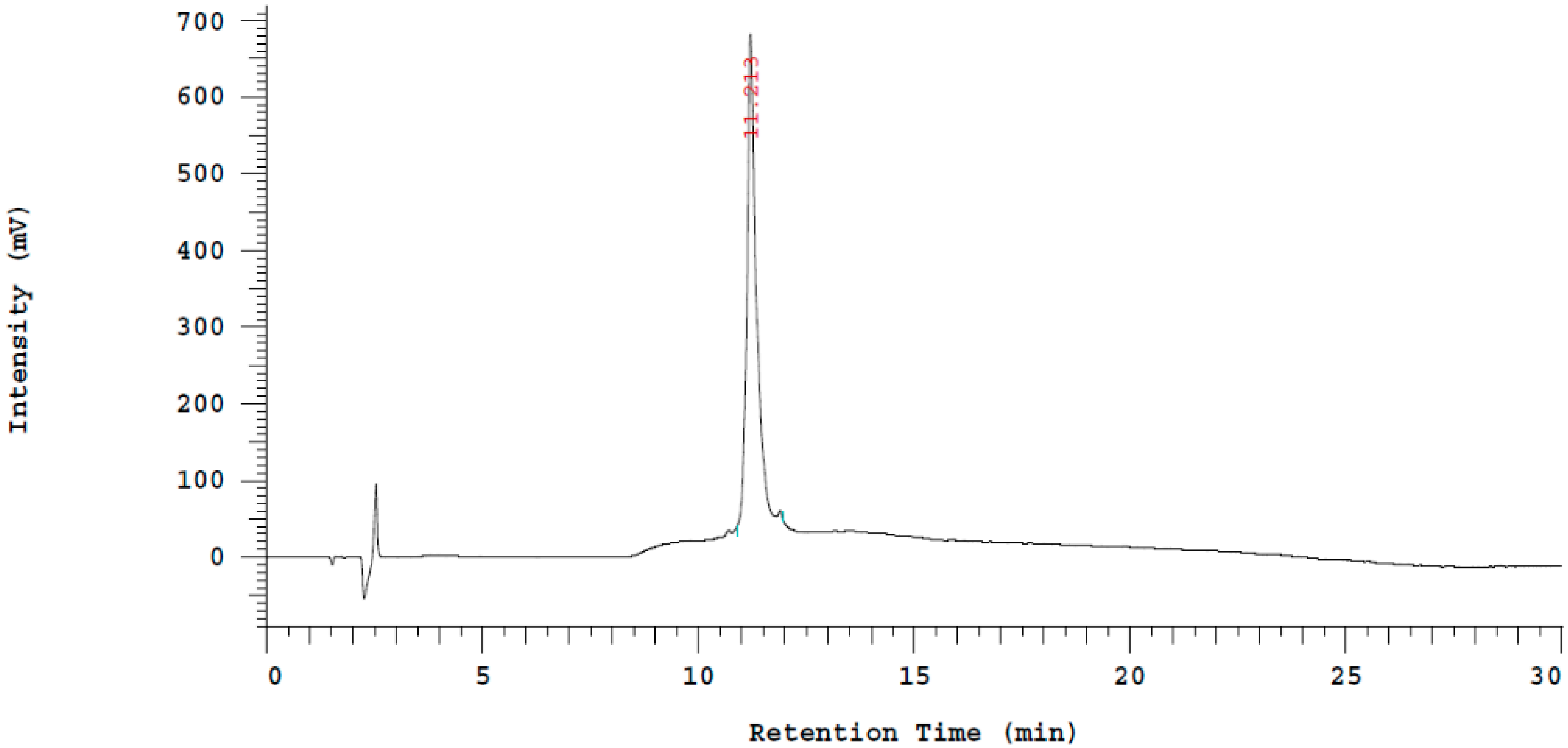

Figure S16. RP-HPLC of PAS_4 (#1094).

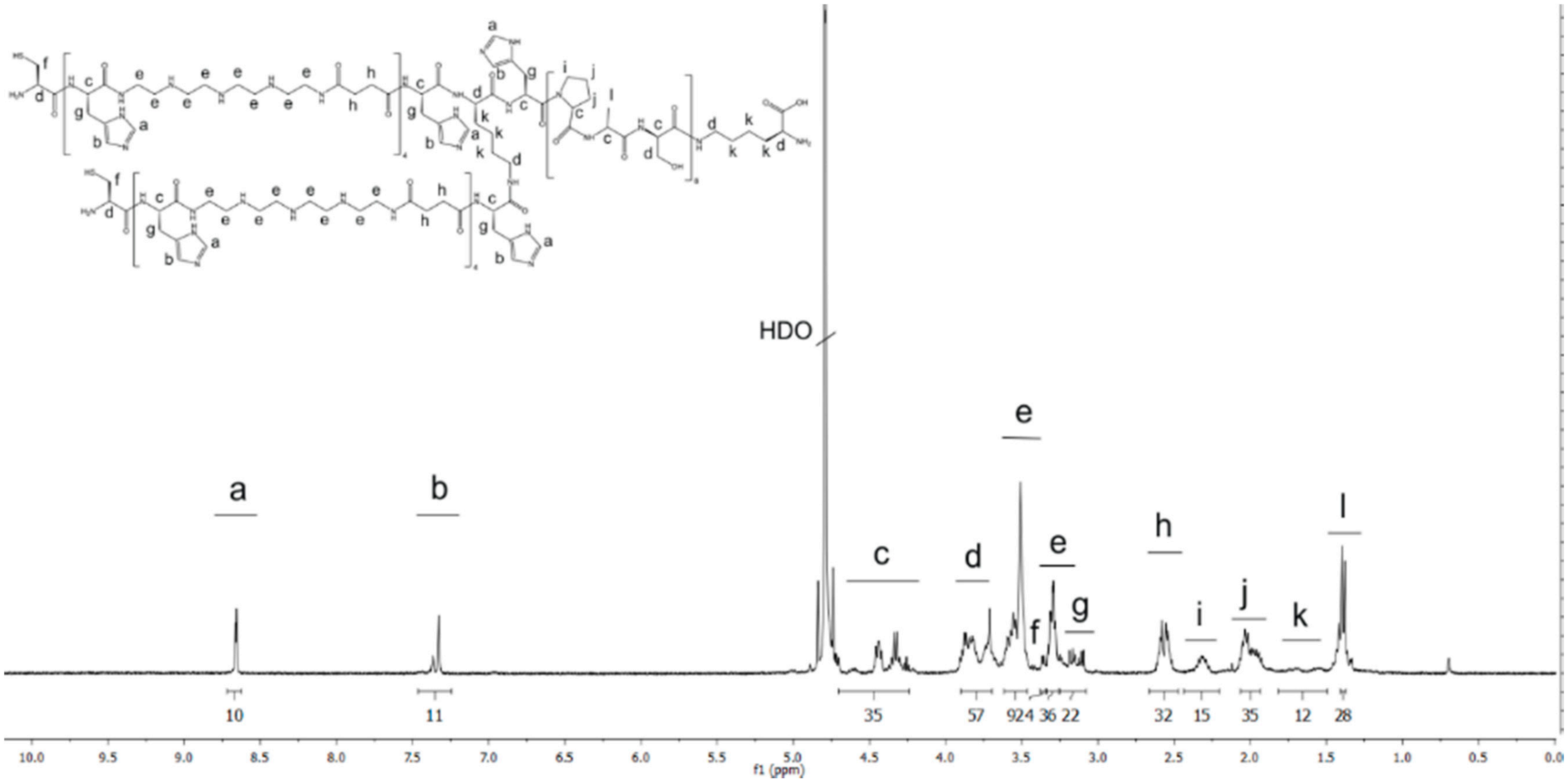

Figure S17. ^{1}H-NMR spectrum of PAS_8 (#1097) in D_2O. Sequence (N–>C): $\{[C–(H–Stp)_4–H]_{\alpha,\varepsilon}–K–H–(PAS)_8\}_\varepsilon–K$.δ (ppm) = 1.3-1.4 (td, 24H, $-CH_3$ H alanine), 1.45-1.9 (comp, 12H βγδH lysine), 1.95-2.45 (comp, 48H, -CH2 proline), 2.5-2.7 (comp, 32 H, $-CO-CH_2-CH_2-CO-$ succinic acid), 3.1-3.65 (comp, 154 H, $-CH_2-$ tepa, βH cysteine, βH histidine), 3.70-3.90 (m, 24H, αεH lysine, βH serine, αH cysteine) 4.1-4.7 (comp, 35 H, αH histidine, αH serine, αH proline, αH alanine), 4.79 (s, HDO), 7.2-7.4 (m, 11 H, aromatic H histidine), 8.5-8.7 (m, 11 H, aromatic H histidine). comp indicates a group of overlaid protons.

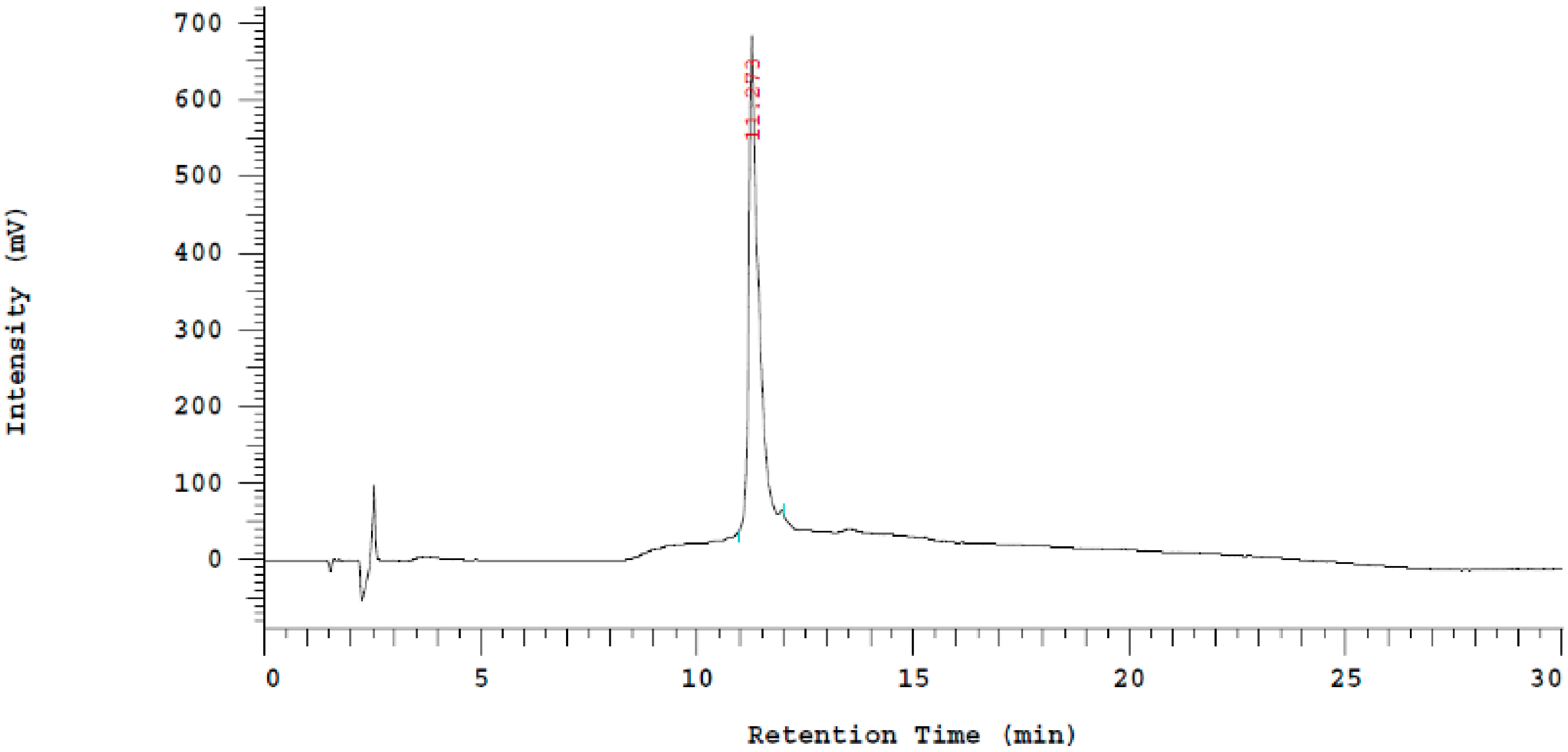

Figure S18. RP-HPLC of PAS_8 (#1097).

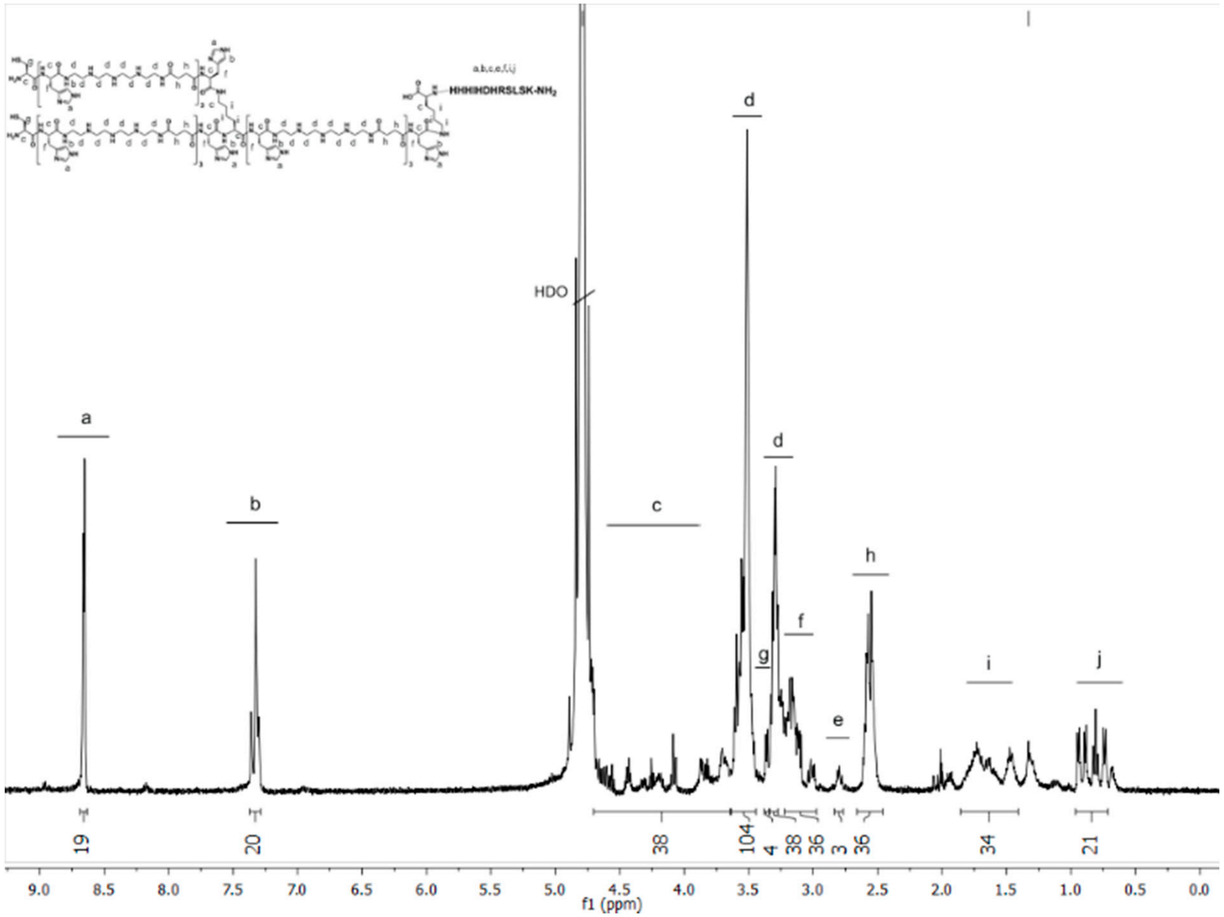

Figure S19. ^{1}H-NMR spectrum of cmb-3-arm (#1078) in D_2O. Sequence (N–>C): KSLSRHDHIHHH–[(C–(H–Stp)$_3$–H)$_{\alpha,\varepsilon}$K–H–(Stp–H)$_3$]$_\varepsilon$K. δ (ppm) = 0.7–0.95 (comp, 17H, βγδH leucine, βγδH isoleucine), 1.35–1.8 (comp, 24H, βγδH lysine, βγδH arginine), 2.3–2.7 (comp, 36 H, –CO–CH_2–CH_2–CO– succinic acid), 2.6–2.85 (t, 2 H, asparagine), 2.95–3.2 (comp, 34 H, β histidine) 3.25–3.6 (comp, 150 H, –CH_2– tepa, βH cysteine), 3.65–4.7 (comp, 16 H, αH amino acids, βH serine, εH lysine), 4.79 (s, HDO), 7.2–7.4 (m, 17 H, aromatic H histidine), 8.5–8.7 (m, 17 H, aromatic H histidine). Comp indicates a group of overlaid protons.

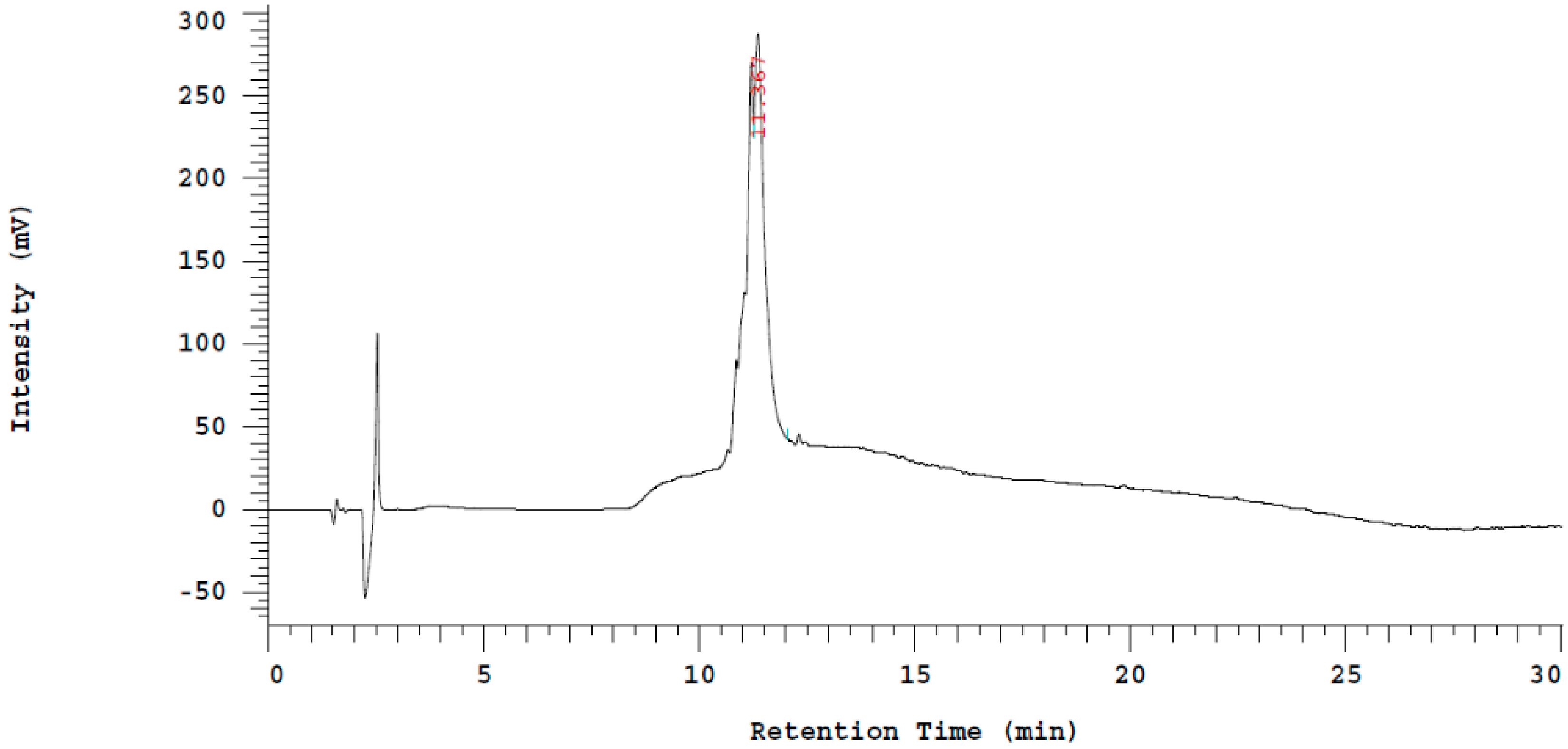

Figure S20. RP-HPLC of cmb-3-arm (#1078).

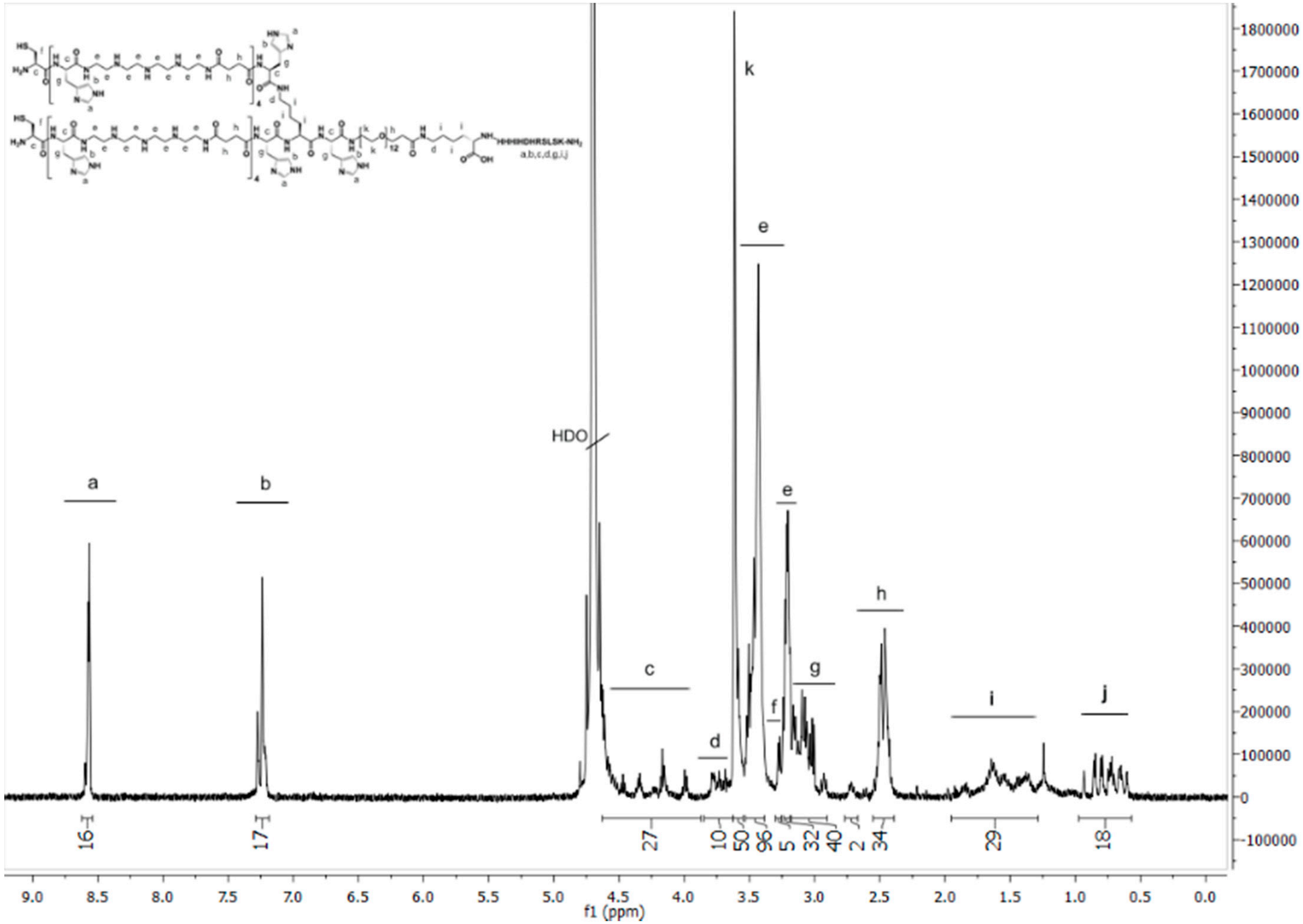

Figure S21. ^{1}H-NMR spectrum of cmb-PEG$_{12}$ (#996) in D_2O. Sequence (N–>C): KSLSRHDHIHHH–[(C–(H–Stp)$_4$–H)$_{\alpha,\varepsilon}$K–H–dPEG$_{12}$]$_\varepsilon$K.δ (ppm) = 0.7–0.95 (comp, 17H, βγδH leucine, βγδH isoleucine), 1.35–1.8 (comp, 24H, βγδH lysine, βγδH arginine), 2.3–2.7 (comp, 34 H, –CO–CH_2–CH_2–CO– succinic acid), 2.45–2.55 (t, 2 H, asparagine), 2.8–3.15 (comp, 32 H, β histidine) 3.20–3.52 (comp, 132 H, –CH_2– tepa, βH cysteine), 3.6 (s, 50H, –CH2–O–dPEG$_{12}$) 3.65–4.7 (comp, 37 H, αH amino acids, βH serine, εH lysine), 4.79 (s, HDO), 7.2–7.3 (m, 16 H, aromatic H histidine), 8.5–8.7 (m, 16 H, aromatic H histidine). comp indicates a group of overlaid protons.

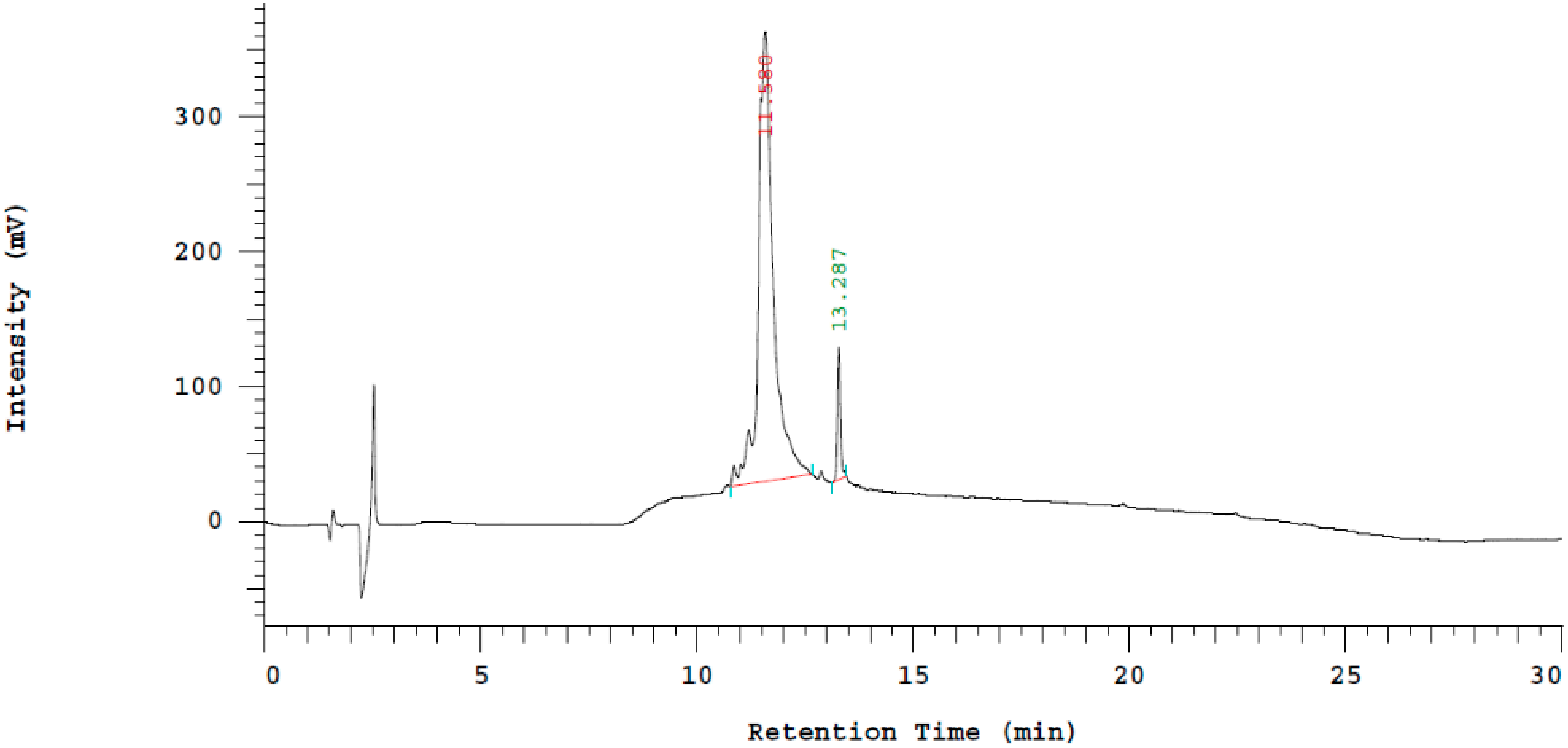

Figure S22. RP-HPLC of cmb-PEG$_{12}$ (#996).

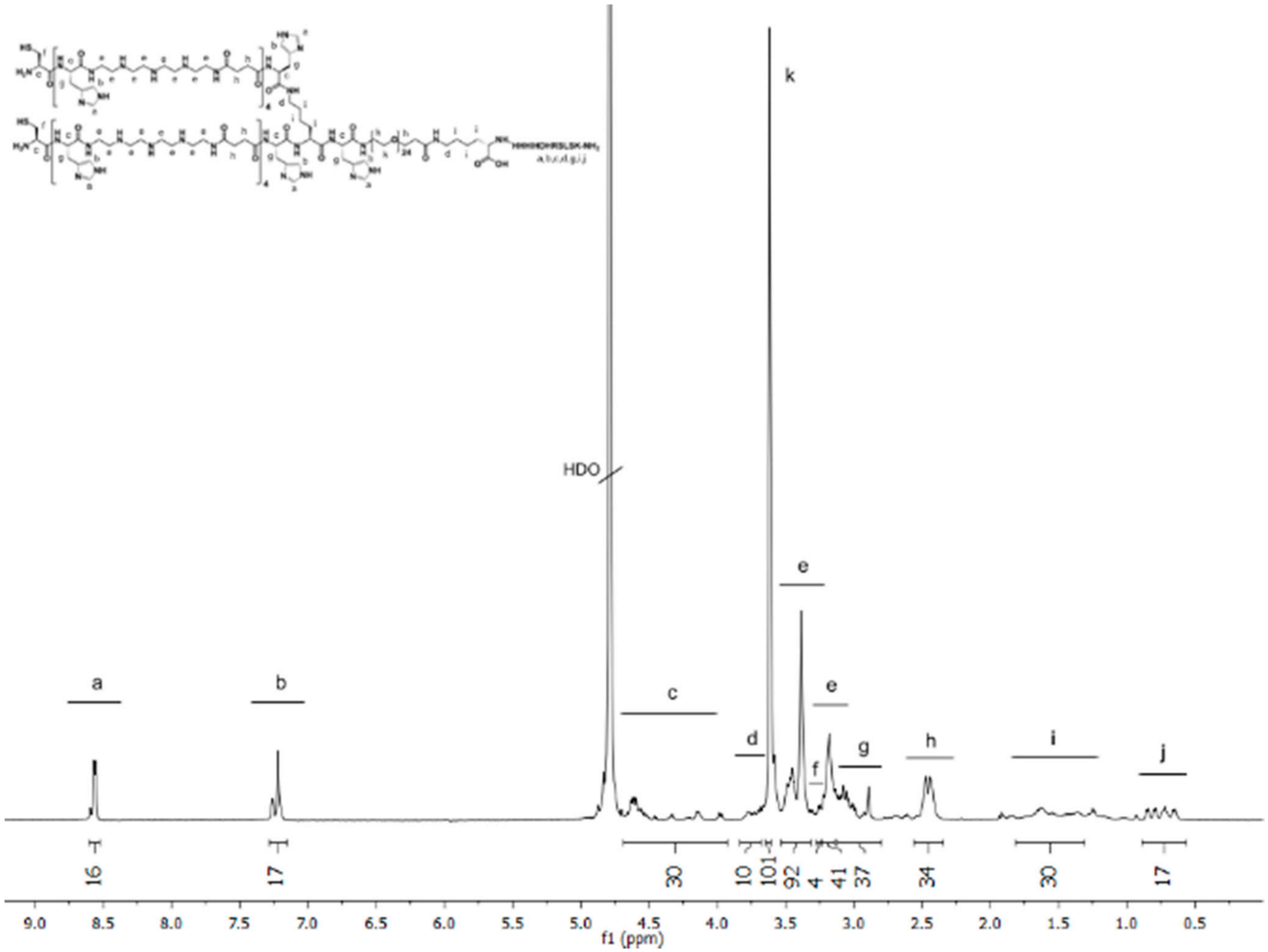

Figure S23. ^{1}H-NMR spectrum of cmb-PEG24 (#442) in D_2O. Sequence (N–>C): KSLSRHDHIHHH–[(C–(H–Stp)$_4$–H)$_{\alpha,\varepsilon}$K–H–dPEG$_{24}$]$_\varepsilon$K. δ (ppm) = 0.7–0.95 (comp, 17H, βγδH leucine, βγδH isoleucine), 1.35–1.8 (comp, 24H, βγδH lysine, βγδH arginine), 2.3–2.7 (comp, 34 H, –CO–CH_2–CH_2–CO–succinic acid), 2.8–3.15 (comp, 34 H, βH histidine, βH asparagine) 3.20–3.55 (comp, 132 H, –CH_2– tepa, βH cysteine), 3.6 (s, 98H, –CH2–O–dPEG$_{24}$) 3.65–4.7 (comp, 37 H, αH amino acids, βH serine, εH lysine), 4.79 (s, HDO), 7.2–7.3 (m, 16 H, aromatic H histidine), 8.5–8.7 (m, 16 H, aromatic H histidine). Comp indicates a group of overlaid protons.

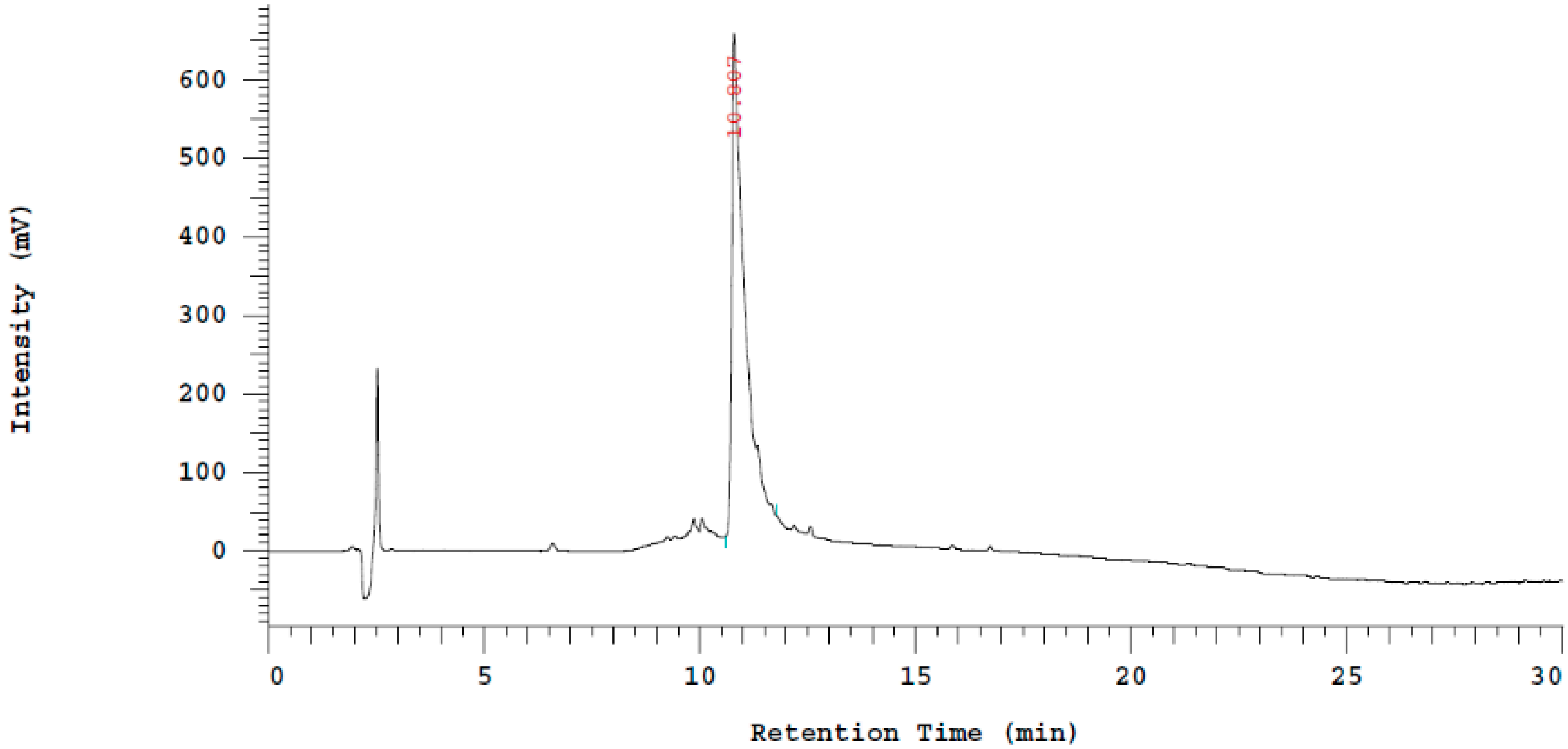

Figure S24. RP-HPLC of cmb-PEG$_{24}$ (#442).

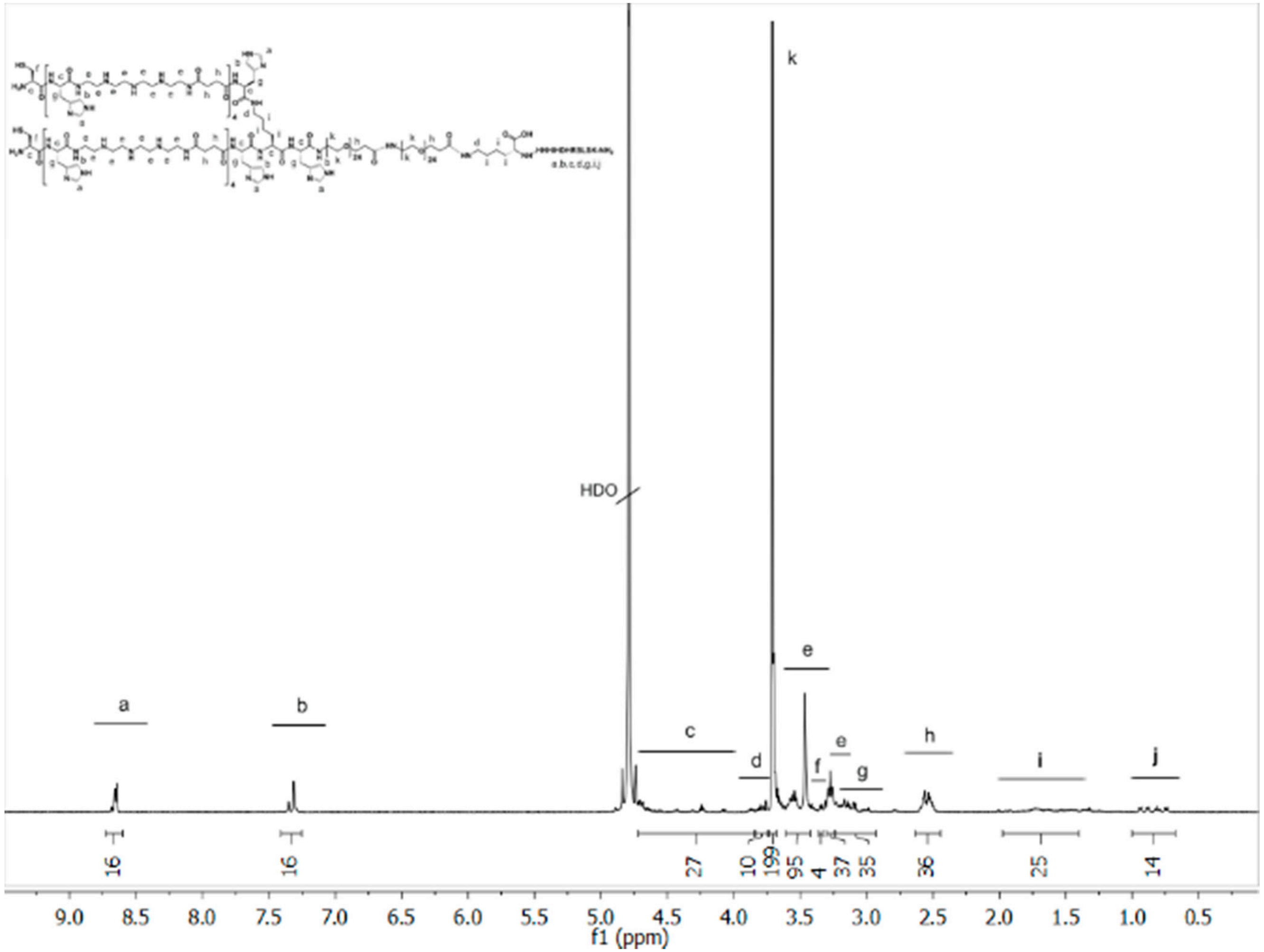

Figure S25. ^{1}H-NMR spectrum of cmb-PEG$_{48}$ (#694) in D_2O. Sequence (N–>C): KSLSRHDHIHHH–[(C–(H–Stp)$_4$–H)$_{\alpha,\varepsilon}$K–H–dPEG$_{24}$-dPEG$_{24}$]$_\varepsilon$K. δ (ppm) = 0.7–0.95 (comp, 17H, βγδH leucine, βγδH isoleucine), 1.35–1.8 (comp, 24H, βγδH lysine, βγδH arginine), 2.3–2.7 (comp, 36 H, –CO–CH_2-CH_2-CO- succinic acid), 2.8–3.15 (comp, 36H, βH histidine, βH asparagine) 3.20–3.55 (comp, 132H, –CH_2– tepa, βH cysteine), 3.7 (s, 196H, –CH2–O–dPEG$_{24}$) 3.72–4.7 (comp, 37 H, αH amino acids, βH serine, εH lysine), 4.79 (s, HDO), 7.2–7.3 (m, 16 H, aromatic H histidine), 8.5-8.7 (m, 16H, aromatic H histidine). comp indicates a group of overlaid protons.

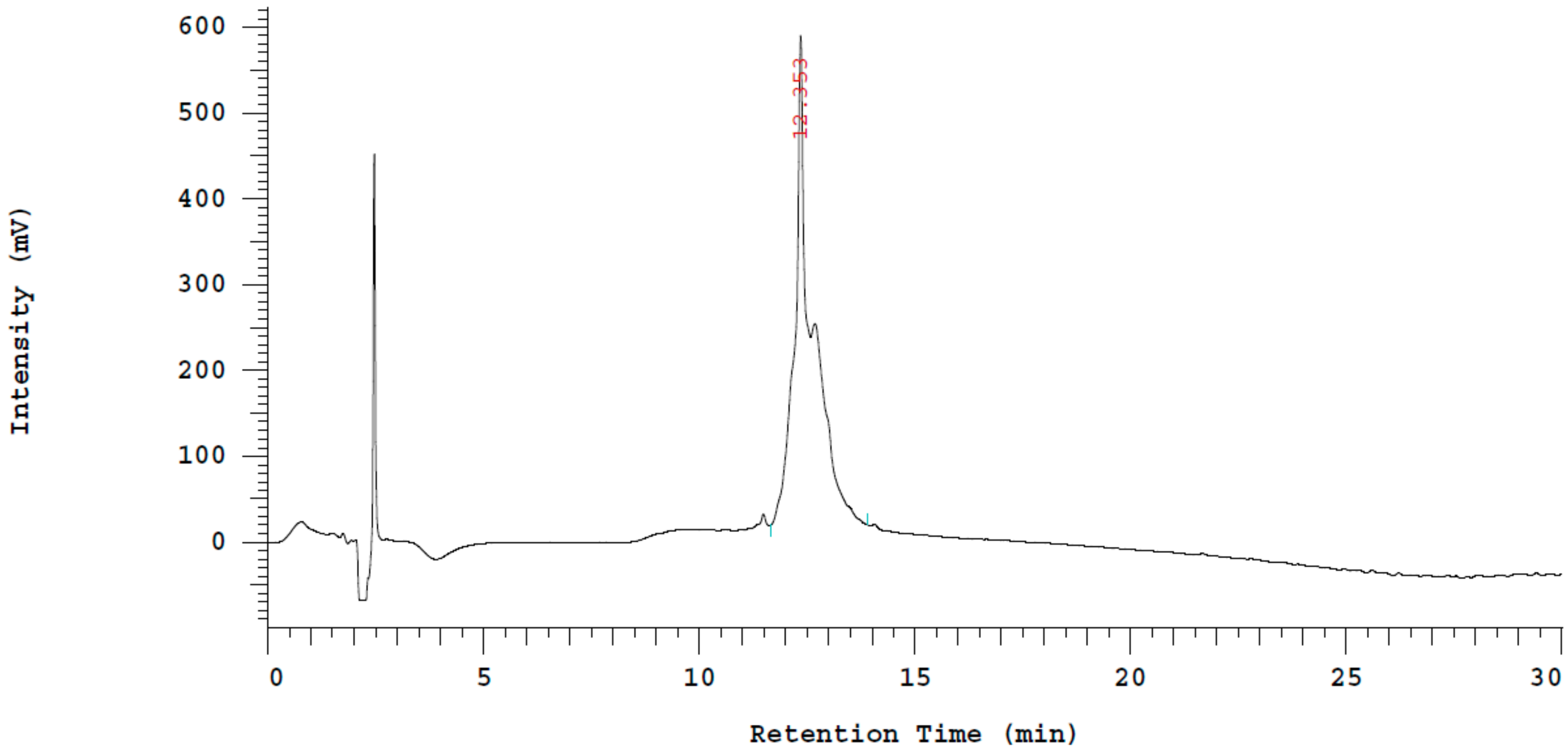

Figure S26. RP-HPLC of cmb-PEG48 (#694).

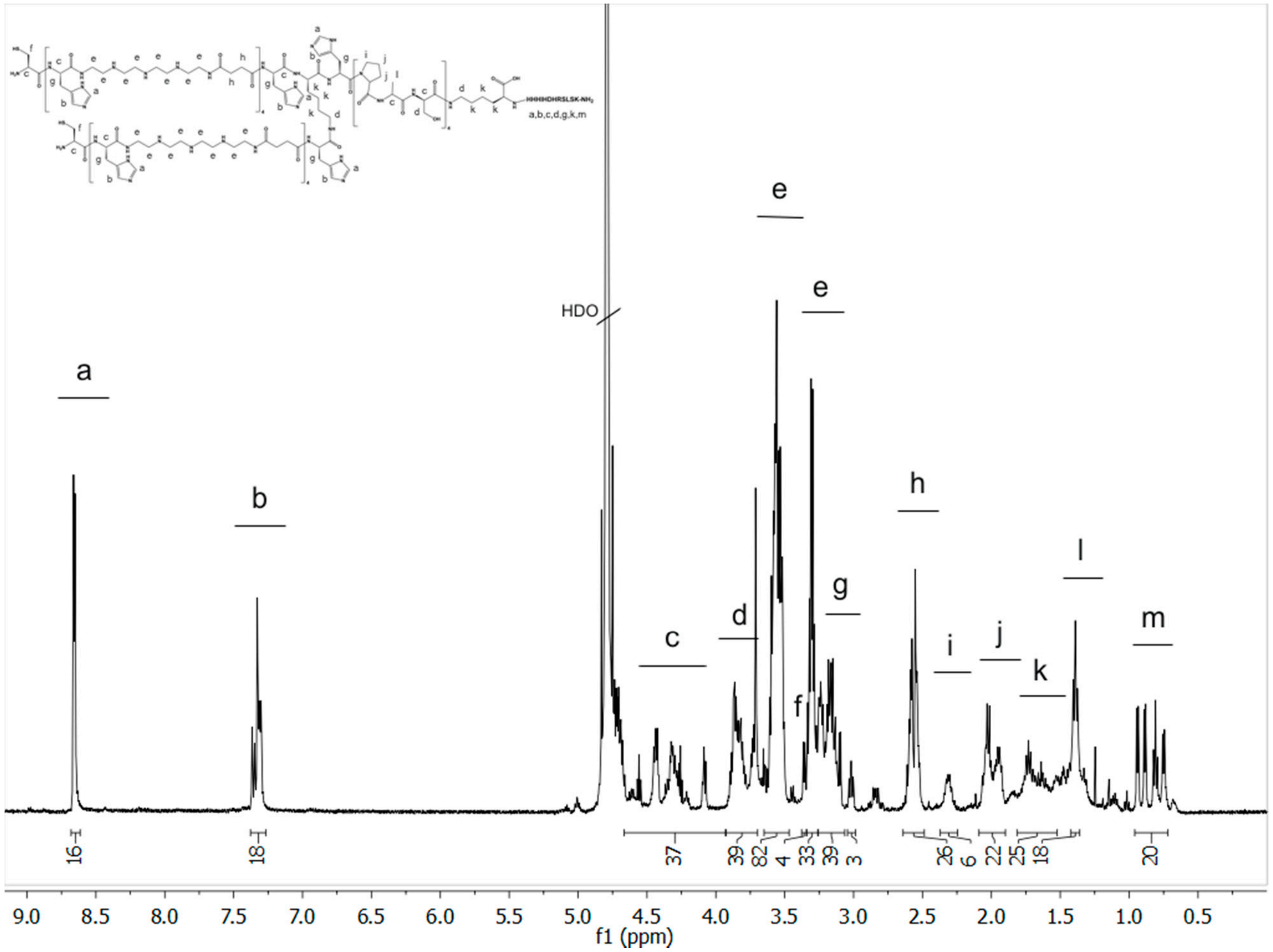

Figure S27. ^{1}H-NMR spectrum of cmb-PAS$_4$ (#1000) in D_2O. Sequence (N->C): KSLSRHDHIHHH-[(C-(H-Stp)$_4$-H)$_{\alpha,\varepsilon}$K-H-(PAS)$_4$]$_\varepsilon$K. δ (ppm) = 0.7–0.95 (comp, 17H, βγδH leucine, βγδH isoleucine), 1.3–1.4 (td, 12H, -CH_3 H alanine), 1.5–1.9 (comp, 24H, βγδH lysine, βγδH arginine), 1.95–2.45 (comp, 24H, –CH2 proline), 2.5–2.7 (comp, 36H, –CO–CH_2–CH_2–CO– succinic acid), 3.0 (t, 2 βH, asparagine), 3.15–3.3 (comp, 34H, βH histidine), 3.3–3.6 (comp, 132 H, –CH_2– tepa, βH cysteine), 3.75–4.7 (comp, 57H, βH serine, εH lysine, αH amino acids), 4.79 (s, HDO), 7.3–7.4 (m, 16 H, aromatic H histidine), 8.6–8.8 (m, 16 H, aromatic H histidine). comp indicates a group of overlaid protons.

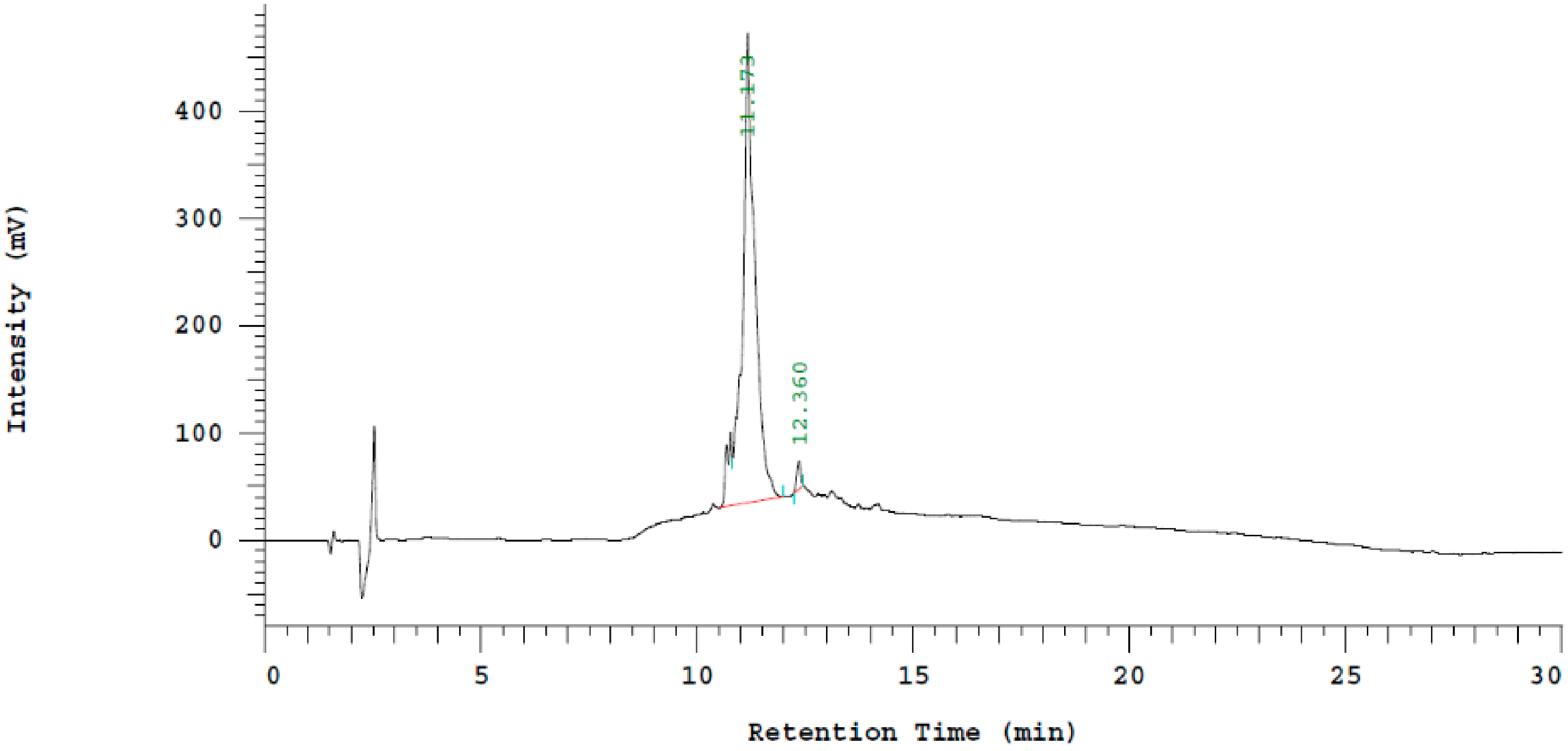

Figure S28. RP-HPLC of cmb-PAS$_4$ (#1000).

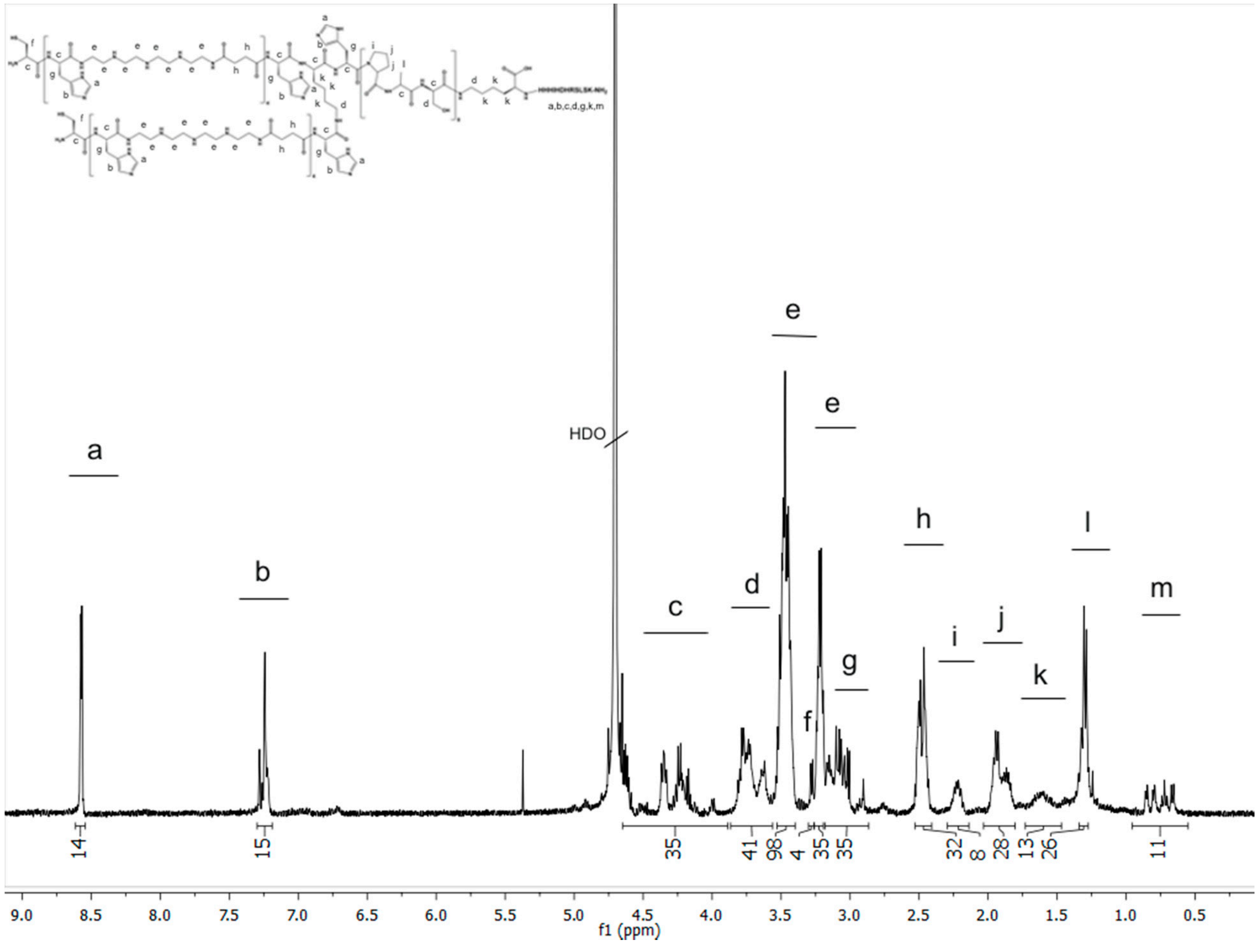

Figure S29. ^{1}H-NMR spectrum of cmb-PAS$_8$ (#901) in D_2O. Sequence (N–>C): KSLSRHDHIHHH–[(C–(H-Stp)$_4$–H)$_{\alpha,\varepsilon}$K–H–(PAS)$_8$]$_\varepsilon$K. δ (ppm) = 0.7–0.95 (comp, 17H, βγδH leucine, βγδH isoleucine), 1.3–1.4 (td, 24H, –CH_3–H alanine), 1.5–1.9 (comp, 24H, βγδH lysine, βγδH arginine), 1.75–2.25 (comp, 48H, –CH2 proline), 2.5–2.7 (comp, 32H, –CO–CH_2–CH_2–CO– succinic acid), 2.8–3.25 (comp, 34H, βH histidine, βH asparagine), 3.3–3.6 (comp, 132 H, –CH_2– tepa, βH cysteine), 3.75–4.7 (comp, 77H, βH serine, εH lysine, αH amino acids), 4.79 (s, HDO), 7.25–7.4 (m, 16 H, aromatic H histidine), 8.6–8.65 (m, 16 H, aromatic H histidine). Comp indicates a group of overlaid protons.

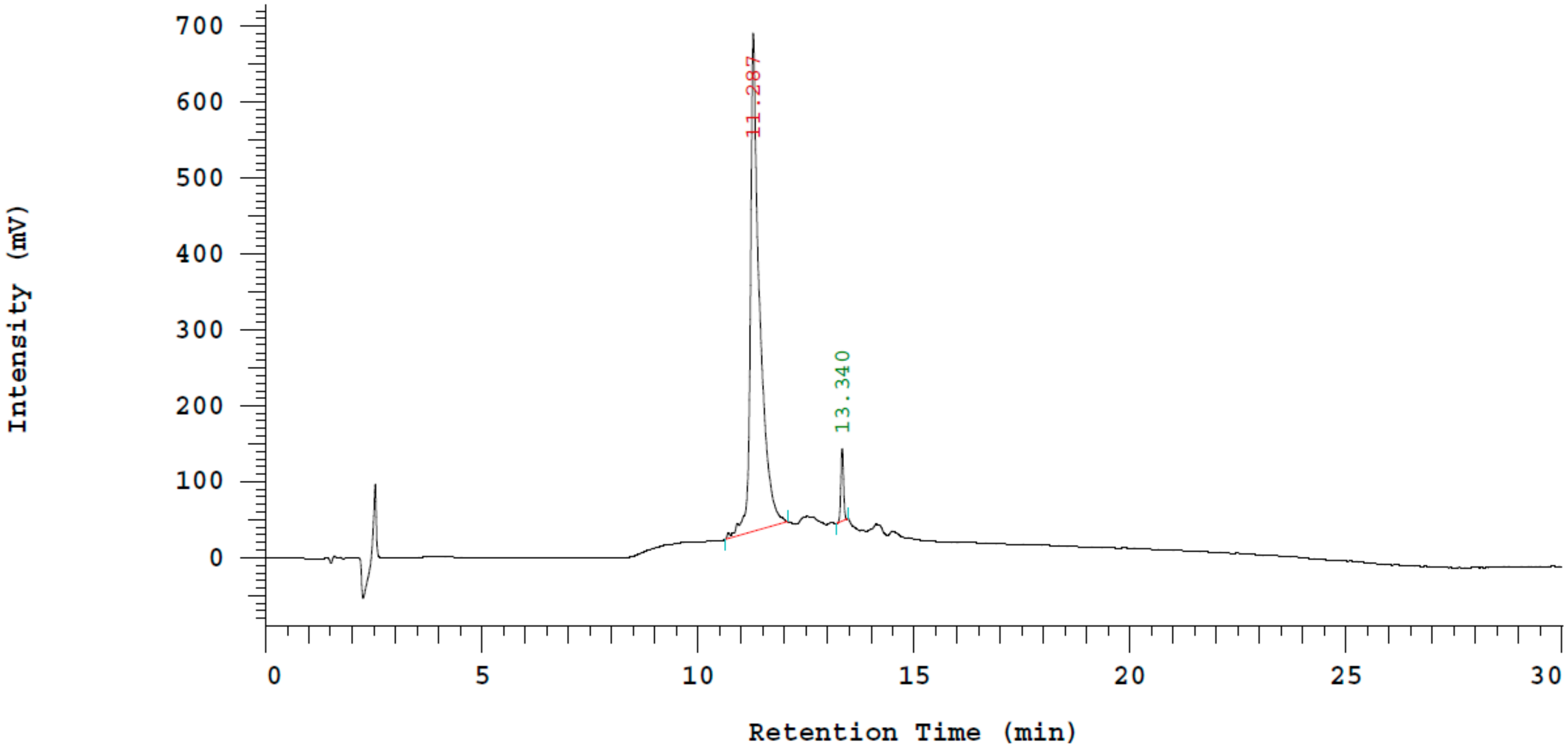

Figure S30. RP-HPLC of cmb-PAS8 (#901).

References

1. Kaiser, E.; Colescott, R.L.; Bossinger, C.D.; Cook, P.I. Color test for detection of free terminal amino groups in the solid-phase synthesis of peptides. *Anal. Biochem.* **1970**, *34*, 595–598.
2. Morys, S.; Wagner, E.; Lachelt, U. From artificial amino acids to sequence-defined targeted oligoaminoamides. *Methods Mol. Biol.* **2016**, *1445*, 235–258.
